# Evaluating Potential Risks of Food Allergy and Toxicity of Soy Leghemoglobin Expressed in *Pichia pastoris*

**DOI:** 10.1002/mnfr.201700297

**Published:** 2017-10-17

**Authors:** Yuan Jin, Xiaoyun He, Kwame Andoh‐Kumi, Rachel Z. Fraser, Mei Lu, Richard E. Goodman

**Affiliations:** ^1^ Food Allergy Research and Resource Program Dept. of Food Science & Technology University of Nebraska‐Lincoln Lincoln NE USA; ^2^ College of Food Science and Nutritional Engineering China Agricultural University Beijing China; ^3^ Impossible Foods Inc. Redwood City CA USA

**Keywords:** allergenicity, bioinformatics, GMO, hemoglobin, toxicity

## Abstract

**Scope:**

The Soybean (*Glycine max*) leghemoglobin c2 (LegHb) gene was introduced into *Pichia pastoris* yeast for sustainable production of a heme‐carrying protein, for organoleptic use in plant‐based meat. The potential allergenicity and toxicity of LegHb and 17 *Pichia* host‐proteins each representing ≥1% of total protein in production batches are evaluated by literature review, bioinformatics sequence comparisons to known allergens or toxins, and in vitro pepsin digestion.

**Methods and results:**

Literature searches found no evidence of allergenicity or toxicity for these proteins. There are no significant sequence matches of LegHb to known allergens or toxins. Eleven *Pichia* proteins have modest identity matches to minor environmental allergens and 13 *Pichia* proteins have significant matches to proteins from toxic sources. Yet the matched allergens and toxins have similar matches to proteins from the commonly consumed yeast *Saccharomyces cerevisiae*, without evidence of food allergy or toxicity. The demonstrated history of safe use indicates additional tests for allergenicity and toxicity are not needed. The LegHb and *Pichia sp*. proteins were rapidly digested by pepsin at pH 2.

**Conclusion:**

These results demonstrate that foods containing recombinant soy LegHb produced in *Pichia sp*. are unlikely to present an unacceptable risk of allergenicity or toxicity to consumers.

## 1 Introduction

Hemoglobins (Hbs) are ubiquitous iron binding proteins in nature, present in bacteria, fungi, higher plants, and animals.^1^ Consumption of these proteins serves as an efficient source of bioavailable iron, which is required for oxygen transport, respiration, and other metabolic functions.^2^ Animal Hbs and myoglobins have long been widely consumed in the human diet through meat, poultry, and fish products. Dietary exposure to plant Hbs is also common. Sprouted barley, widely used in the beverage industry (malted barley) and in the baking industry (malted barley flour), has been shown to express Hb 2–3 days after imbibition.^3^ In legumes, four major leghemoglobin (LegHb) isoproteins are generally found in root nodules, where atmospheric N_2_ is reduced to ammonia and assimilated in exchange for photosynthetically produced sugars.^4^, ^5^ LegHb is structurally similar to the widely consumed mammalian myoglobins.^6^ They share an identical heme cofactor (heme B), which binds oxygen with high affinity. These mammalian myoglobins share a common history of safe use in foods.

Impossible Foods Inc. (Redwood City, CA) discovered that heme proteins are critical to the flavor profile of animal‐derived meat (US patent 14/797,006, allowed March 22, 2017). Therefore, to recreate the flavor profile of animal‐derived meat in plant‐based meat products, Impossible Foods synthesized the LegHb c2 gene from the soybean plant, inserted the gene into a yeast host, *Pichia pastoris* (now reclassified as *Komagataella phaffii*), and purified the LegHb protein expressed in the resulting *Pichia* strain to >65% purity. The LegHb protein, produced in this way, has been tested in plant‐based meat products at concentrations up to 0.8%. The LegHb c2 gene from soybean (*Glycine max*) root nodules encodes a 145 amino acid (AA) protein sequence listed as accession number P02236 in the UniProt protein database. Seventeen *P. pastoris* proteins, normally expressed by the yeast host, that remain as minor components in the highly enriched LegHb protein product, were identified by Impossible Foods and the UC Davis Genome center using proteomic analysis. Each *Pichia* protein is present at concentrations of less than 0.1% in the plant‐based meat product.

The yeast *P. pastoris* is widely used as a host for recombinant protein expression.^7^ *Pichia* belongs to the same family of yeast (*Saccharomycetaceae*) as several yeast genera widely used in food: *Saccharomyces*, *Torula*, *Yarrowia*, *Dekkera*, and *Brettanomyces*. *Brettanomyces*, a yeast traditionally used in brewing Belgian beers, belongs to the same sub‐family of yeast as *Pichia*—the *Pichiaceae*. Yeast extract (from *S. cerevisiae* and *Torula*) is frequently consumed in substantial quantities in human diets. *Pichia* is used for the GRAS‐approved production of BD16449 phospholipase C, an enzymatic processing aid for degumming vegetable oils (GRN 204). Additionally, *Pichia* is approved as an animal feed source (21 CFR §573.750) and has been used to produce a number of FDA‐approved pharmaceuticals (http://www.pichia.com/science-center/commercilized-products/). However, *Pichia* is most commonly used for expression of secreted recombinant proteins since it produces recombinant proteins more efficiently than does *Saccharomyces*. At present, there are no reports of *Pichia* intracellular host proteins in human food. The potential for allergenicity and toxicity of the final LegHb product and the seventeen most prominent *Pichia* host proteins were evaluated for safety as food ingredients.

The primary concerns for food safety from any recombinantly expressed protein (REP) are whether the protein itself is allergenic, whether the protein is cross‐reactive due to similarity to another protein, whether the protein is a toxin, or whether insertion of the gene alters the quantity of endogenous allergens if the host is commonly allergenic.^8^, ^9^ The CODEX Alimentarius Commission recommended a weight‐of‐evidence approach to judge whether the introduced novel protein has allergy risk or not.^10^, ^11^ The approach involves a multi‐step process, including review of the allergenicity, or safety of the gene source, sequence similarity comparison of the protein to known allergens, and testing the stability of the protein to digestion by pepsin. Understanding the abundance of the protein in the food products is relevant as well. Since many allergens and toxins have now been identified, a bioinformatic sequence comparison combined with a literature review serves as an important tool for assessing the potential risks.^11^, ^12^ If a significant sequence identity match is found, serum IgE tests using samples from subjects sensitized to the source may be needed for evaluation of the allergenic potential.^12^ The potential toxicity assessment recommended by CODEX includes a review of toxins or toxicity of the source organism and a comparison of the protein AA sequence identity between the novel protein and known protein toxins.^13^ Tests to measure the stability of the protein in an in vitro gastric digestion model are also used to judge potential risks of potential allergenicity or toxicity of the protein.^10^, ^11^, ^12^

Allergenic cross‐reactivity is difficult to predict. Aalberse suggested that proteins with <50% identity in AA sequence over full length with an allergen are unlikely to be cross‐reactive.^14^ However, CODEX recommended using criteria for matches of >35% identity over 80 AA by FASTA as a conservative prediction for potential cross‐reactivity. Although not stated in the guideline, the 80 AA‐segment match is apparently based on the rationale that either natural or man‐made genetic changes might transfer a structural motif that could include sequential or conformational IgE‐binding epitopes capable of cross‐linking IgE on receptors on sensitized mast cells or basophils that could induce allergic mediator release.^11^, ^15^ Some regulatory authorities require a search comparison for eight AA segment identity matches to allergens to identify possible cross‐reactive targets.^10^, ^11^

Resistance to pepsin digestion was recommended by CODEX as a central part of the allergenicity assessment of recombinant proteins intended for human consumption based on earlier publication by Metcalfe et al.^16^ The digestion assay is not meant to predict whether a given protein will always be digested in the stomach of human consumers, but the evaluation provides a simple in vitro correlation between resistance to pepsin digestion and allergenic potential.^17^, ^18^, ^19^

Evaluation of the safety of recombinant LegHb protein and 17 minor yeast proteins were evaluated by literature review, bioinformatic analysis for sequence similarity to proteins in the AllergenOnline (AOL) and NCBI databases, as well as in vitro pepsin digestion by simulated gastric fluid (SGF) at two pepsin–protein ratios, 10:1 and 1:1 (U μg^–1^).

## 2 Experimental Section

### 2.1 Scientific Literature Review

Four public literature databases were used to search for publications indicating possible risk using combinations of keywords (soybean or *Glycine max* with Hb and allergy, allergen, toxic, or toxin as well as the 17 high *Pichia pastoris* proteins) to identify articles that might indicate potential allergenicity or toxicity of the protein or the host organism. These four databases were PubMed (http://www.ncbi.nlm.nih.gov/pubmed), Web of Science™ Core Collection (v.5.24] (http://apps.webofknowledge.com/), Scopus (https://www.scopus.com/), and National Agricultural Library Catalog (AGRICOLA, https://agricola.nal.usda.gov/). The original searches with PubMed were conducted on 07 March 2016 and 01 July 2016 (LegHb and the 17 Pichia proteins, respectively). These searches were repeated with PubMed, adding the Web of Science, Scopus, and Agricola databases on 22 May, 2017. Publication abstracts were reviewed and identified publications searched for indications of potential indications of adverse health risks.

### 2.2 Sequence Databases and Bioinformatics Search Strategies

#### 2.2.1 Sequence of LegHb and the 17 *Pichia* Host Proteins

**Figure 1:**
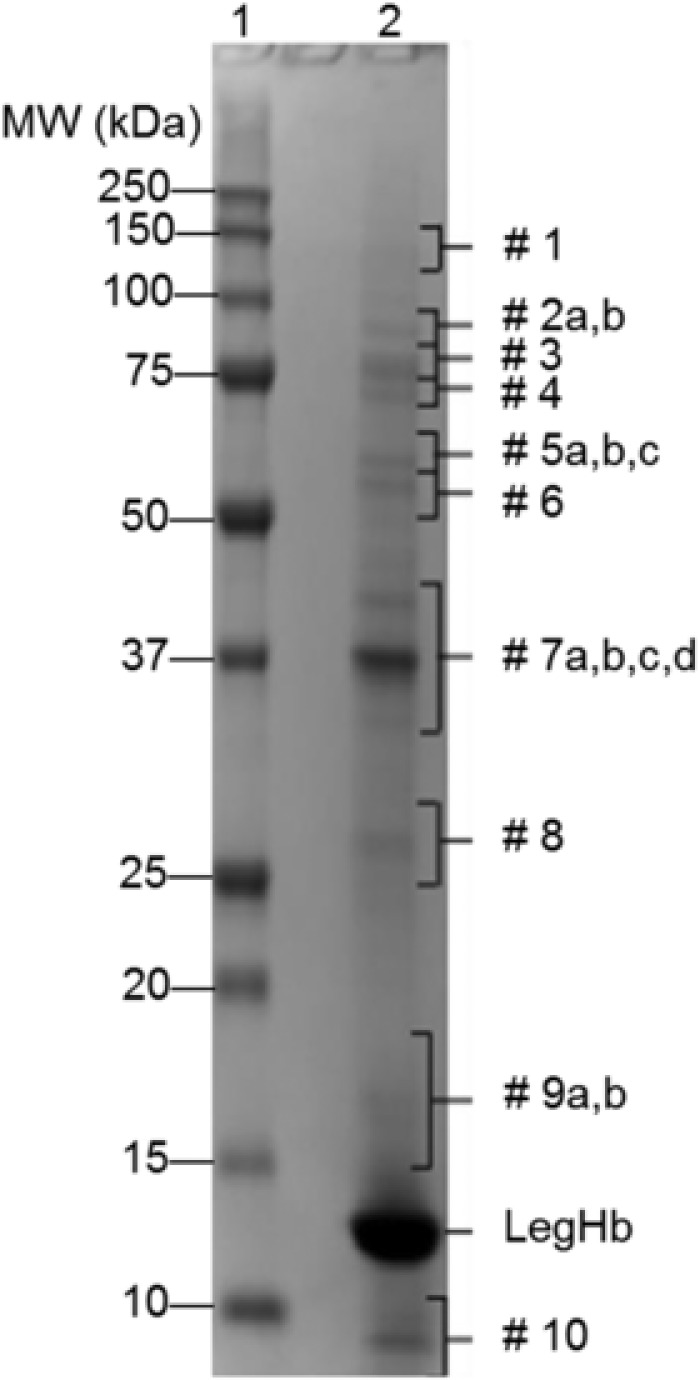
Coomassie Brilliant Blue Stained SDS‐PAGE Gel showing a production lot of LegHb that includes the 17 identified residual *Pichia pastoris* proteins. A production batch of LegHb was applied to an SDS‐PAGE gel using reducing conditions. The MW of the marker proteins (lane 1) are indicated as is the position of LegHb (lane 2) and the major bands from the *Pichia* host.

**Table** 1 shows the full AA sequence of the LegHb protein. **Figure** 1 shows a Coomassie stained SDS‐PAGE gel image of a production scale batch of Soy LegHb preparation (PP‐PGM2‐15‐320‐101), provided by Impossible Foods Inc. Ten visible bands in addition to LegHb were evaluated by LC–MS/MS and found to contain 17 *Pichia* proteins, each band representing ≥1% of the total protein fraction of LegHb preparation based on densitometry. Each protein sequence was used to identify full‐length matches to sequences in the NCBI database (Table S1, Supporting Information). Those sequences were then used as query sequences in bioinformatics searches.

**Table 1:** AA sequence of the soybean LegHb protein produced in *Picchia pastoris*.

Organism	Hb class GI#:	Native protein sequence
	Accession#	
*Glycine max*	LegHb c2 GI:126241 Acc: P02236.2	MGAFTEKQEALVSSSFEAFKANIPQYSVVFYTSILEKAPAAKDLFSFLSNGVDPSNPKLTGHAEKLFGLVRDSAGQLKANGTVVADAALGSIHAQKAITDPQFVVVKEALLKTIKEAVGDKWSDELSSAWEVAYDELAAAIKKAF

#### 2.2.2 AllergenOnline Version 2016 (v16)

The AOL database (http://www.allergenonline.org/) is a peer‐reviewed allergen list and sequence database that is updated each year.^20^ We used the AOL as the primary tool in searching for matches to allergenic sequences. Three comparison methods were used: an overall FASTA3 full‐length search (using *E* scores of 10 and 1.0), a sliding 80 AA window FASTA search (*E* score of 10, identity > 35%), and an exact word search tool looking for eight AA exact matches. The *E* score criteria limits have been established on the AOL database for more than 5 years based on extensive testing to limit irrelevant matches while identifying alignments that may cause cross‐reactivity.^20^

#### 2.2.3 BLASTP in NCBI Entrez Protein Database

The BLASTP search algorithm is available on the NCBI website (http://www.ncbi.nlm.nih.gov/BLAST/). Protein sequence entries in the Entrez search and retrieval system are compiled and maintained by the NCBI of the National Institutes of Health (U.S.A.). The database is updated or modified every few days. Sequences are annotated with publications or with notation of likely protein types; much of that is added by computer algorithm and some simply suggest minor similarities to a previously identified allergen type. Since AOL is updated once per year, the NCBI protein database was searched using keyword limits (“allergen”, “toxin”, and “toxic”) to identify possible matches to proteins that may represent newly discovered allergens or toxins. The searches for matches between newly deposited proteins and the LegHb and the 17 host proteins, respectively, were performed on Feb 27 to 29, 2016 and May 27, 2016, using the BLASTP version 2.3.0. Searches were also performed for each sequence without any keyword to evaluate whether similar proteins exist in other organisms that might provide information of safe exposure to homologous proteins. The additional searches ensured that sequences, which might not yet have been entered into AOL v16, were included in the analysis.^21^ Additional bioinformatics searches with NCBI and BLASTP were used to compare sequence identity ranking with proteins of risk or from known safe food sources. We evaluated the *E* score values, the length of the alignments, and the identity percentage of any identified match from the BLASTP results to judge the significance of any alignment.

### 2.3 In Vitro Pepsin Digestibility Study

The test system for this study was an in vitro digestion model using pepsin in SGF. The SGF preparation and digestion procedures were based on the methods described by Thomas et al.,^22^ as modified by Ofori‐Anti.^18^ Pepsin is optimally active between pH 1.2 and 2.0, but markedly less active at pH 3.5, and irreversibly denatured at pH 7.0.^23^, ^24^ Although the first pepsin digestion assay developed by Astwood recommended performing the digestion at pH 1.2,^25^ the FAO/WHO^26^ suggested using two pH conditions, pH 1.2 and 2.0 (FAO/WHO).^26^ By comparing pH 2.0 versus 1.2, Thomas et al.^22^ showed that protein digestion at pH 2.0 resulted in slightly slower rates of full‐length protein and fragment degradation, but did not alter the overall sensitivity of a protein to digestion.^22^ Moreover, we digested a number of proteins at both pH 1.2 and 2.0 and did not demonstrate significant differences.^18^ Therefore, in this study we only evaluated stability of the protein at pH 2.0.

#### 2.3.1 Materials

##### 2.3.1.1 Test and Control Substances

The protein sample solution was provided by Impossible Foods Inc. from the LegHb production run PP‐PGM2‐15‐320‐101. The LegHb protein represented 66% of the total protein according to the certificate of analysis. The total protein concentration was determined to be 79.94 mg mL^–1^ using a GE 2D Quant kit (GE Healthcare, #80‐6483‐56), following the kit instructions. The PP‐PGM2‐15‐320‐101 is a representative batch of soy LegHb protein preparation. The type and abundance of *Pichia* proteins is consistent from batch‐to‐batch. Bovine Hb (Sigma–Aldrich Co. LLC., St. Louis, MO, #H2625‐25G), BSA (Sigma‐Aldrich Co. LLC., MO #A9647‐100G), and chicken ovalbumin (OVA; Worthington Biochemicals, #3054) were used as control proteins in the digestion assay.

Pepsin A (Worthington Biochemicals, #3319) with a certificate of analysis of 2810 activity units per mg solid was used in all assays. Novex® 10–20% tris‐glycine polyacrylamide gels (Invitrogen) were used to separate digested materials. Precision Plus Protein^TM^ dual xtra standards (BioRad) were used as the molecular weight standards. Tris‐Glycine‐SDS 10 × running buffer (Fisher Scientific), Coomassie Brilliant Blue R‐250 staining solution, and Coomassie Brilliant Blue R‐250 destaining solution (BioRad) were used for gel running, staining, and destaining, respectively.

#### 2.3.2 Pepsin Activity Test

The activity of the pepsin was tested within 24 h before each digestion assay. The assay was based on the method described by Worthington, as described by Ofori‐Anti.^18^ Pepsin A tested at 2826 units per mg solid right before the digestion assays, which was quite close to its labeled activity of 2810 U mg^–1^.

#### 2.3.3 Determination of System's LOD

The concentration of the undigested protein in SDS‐PAGE was considered to be 100%. The LOD of the test system must be lower than 10%, as the time for digestion is determined to be when the sample reaches 10% of starting material.^18^ In order to establish the detection limit, a serial dilution of the sample solution was prepared with diluent and 5× reducing Laemmli buffer covering the range representing 200% total protein (2.96 μg) per well down to 2.5% (0.037 μg) per well. Samples were separated in an SDS‐PAGE at a constant 150 V for 90 min, followed by staining with Coomassie blue. The LOD (Figure S1, Supporting Information) was 5% of total protein at 100% loaded in the digestion samples. This level of sensitivity was clearly sufficient to detect 10% residual of LegHb or any other protein in the digest.

#### 2.3.4 Pepsin Digestion Resistance Tests of LegHb and Three Control Proteins

The tests were performed at two different pepsin–protein ratios, 10 units and 1 unit pepsin activity per 1 μg substrate protein. The 10 units assay was described by Ofori‐Anti.^18^ The 1 unit assay was done to experimentally investigate the digestibility of the sample at a much lower pepsin–protein ratio, although this ratio has not been validated yet with many allergenic proteins. The “0 min” digestion sample and the undigested sample at 10% protein concentration were prepared by first quenching pepsin at 95 °C for 10 min, then adding protein solution followed by another 10 min heating to denature the proteins.

## 3 Results

### 3.1 Literature Search Results

A literature review for the history of food safety of LegHb in four databases is summarized in Table S2, Supporting Information. None of the publications implicated any allergenic effects of this protein. Searches with “*Glycine max*”, “allergy”, and “heme” only yielded one reference from Pubmed database on impairment of carotenoid and flavonoid biosynthesis due to a mutation in Arabidopsis HY1, which is not relevant to the topic under review.^27^ A broader search of the literature demonstrates that soybean is reported to be a relatively common food allergen for young children, rarely for adults. Food allergic reactions are to proteins expressed in the seeds. There are no reports of allergy to the other parts of the plant except seed‐pod proteins, referred by as Gly m 1 and Gly m 2 in the WHO/IUIS Allergen Nomenclature database (www.allergen.org/). No reports were identified for root‐specific proteins. Since the LegHb is not present in the seed or seed‐pods but only in root nodules, there is no reason to believe that people would be allergic to the protein and there is no need to perform serum IgE binding studies, and there is no sensitive population to obtain relevant serum for IgE binding studies.

Similarly, with the search filtered for keywords “toxin” or “toxicity”, the majority of the papers reported on the effect of heme‐peroxidases or heme‐oxygenase system and the adverse effect due to changing of hydrogen peroxide level,^28^ which is not relevant to allergenicity or toxicity. Thus, based on literature search, there is no reason to suspect the LegHb produced from *Glycine max* would raise a concern of allergy or toxicity.

A search for literature concerning the safe use of *Pichia pastoris* is summarized in Table S3, Supporting Information. A review of the summary entries showed that they are related to the use of the yeast as a recombinant host for expressing a wide variety of proteins from various eukaryotic sources. In the Pubmed search, adding a third term “NOT recombinant” reduced the findings to five publications.^29^, ^30^, ^31^, ^32^, ^33^ In the other three databases, the numbers were also reduced by adding keyword “NOT recombinant”. Examination of the results demonstrated no link to allergy related to endogenous proteins from *Pichia pastoris*. Searches with the revised name “*Komagataella phaffii*” without other terms greatly reduced the number of the results which were related to taxonomy or genomic cloning of the yeast species (synon. *Pichia pastoris*) or the use of this species as a recombinant host. Full evaluation of the potential risks of allergy of each of the identified 17 *P. pastoris* proteins was considered in searches in combination with “*Pichia pastoris”*. Review of the information failed to identify any study data to suggest possible allergenicity of the endogenous proteins. Thus, there is no published evidence that endogenous proteins from *Pichia pastoris* are allergens.

Searches using a combination of “*Pichia pastoris*” and “toxin” yielded a large number of references in the four databases; most described expression of various eukaryotic genes to produce recombinant proteins. The list was reduced by excluding “recombinant”. One reference from Pubmed specifically examined toxin expression in the host, testing various *P. pastoris* (*Komagataella phaffii*) strains for toxins that are active against pathogens or other yeasts.^34^ They did not detect any evidence of toxic activity in the 14 tested strains. Another study published and identified in Scopus evaluated the Kpkt hertologous protein produced in *P. pastoris*.^35^ This Kpkt protein occurs naturally in a different genus with similar species name, *Tetrapisispora phaffii*. The Kpkt protein is active in reducing microbial spoilage in wine production.^36^ No other publications were identified that were related to toxicity except those from heterologously expressed transgenes from other organisms. Thus, no evidence was found indicating that *P. pastoris* has endogenous toxic proteins.

### 3.2 Bioinformatics Search Results of LegHb

Searches with the LegHb AA sequence against AOL v16 did not identify any significant alignment with an allergen. Full length alignments from FASTA3 searches with *E* scores <1.0 are shown in Table S4, Supporting Information. Only one type of protein, the globin (insect Hb) from *Chironomus thummi thummi*, aligned with <27% identity, which is below a level likely to indicate cross‐reactivity.^14^ Ten additional alignments to taxonomically diverse sources (grass, house dust mite, potato, and cockroach) were identified with *E* scores between 2.2 and eight and are not shown. Neither the sliding 80‐mer window searches nor eight‐contiguous AA search resulted in any matches. Thus, it is unlikely that the LegHb protein is sufficiently similar to any known allergen to present a risk of cross‐reactivity. The lack of sequence identity to known allergens as well as lack of allergy to the source (soybean root nodules), removes the normal requirement for serum IgE testing even though the protein is from soybean, a relatively commonly allergenic source.

The full‐length sequence of LegHb was compared to all sequences in the NCBI–Entrez database without any keyword limit (data not shown). LegHb is highly similar and closely related to other plant Hb proteins that bind oxygen, predominantly in other legumes. The protein buffers the oxygen concentration to enable symbiotic microbes in root nodules to fix nitrogen. LegHb is also approximately 26% identical to some chordate Hbs. The results indicate the high similarity of LegHb to common plant and animal proteins without obvious indications of risks of allergy. BLASTP searches filtered with keywords “allergen” or “toxic” failed to identify matches, while BLASTP with keywords “allergy” or “toxin” produced low identity matches to sequences that are unlikely to be homologous due to low identities or short alignments (Table S5, Supporting Information).

### 3.3 Bioinformatics Search Results of 17 *Pichia* Proteins

Each of the 17 *Pichia* proteins were evaluated for potential matches to allergens in the AOL v16 database. The results are summarized in **Table** 2. Each protein was also compared to the NCBI–Entrez database without any keyword selection, and with keywords “allergen”, “toxin”, or “toxic”. Summaries for each protein are shown in Tables S6–S22, Supporting Information. Overall, these bioinformatics search results showed little risk of food allergy or toxicity.

**Table 2:** Summary of sequence alignments for LegHb and the 17 host proteins identified in AllergenOnline searches.

**Query protein**			**AOL results**							
			**Full FASTA**			**80 mer**			**8 mer**	
			No. of matches			Matched allergend)	Number of hits ID > 35%	Best IDe)	Matched allergen	Number of hits
**Number** a)	**Protein name (GI#) [Accession]**	**Number of AA** b)	*E* score	IDc) >50%						
			<10	<1						
n/a	LegHb (126241) [P02236.2]	145	20+	12	0	n/ae)	0	n/a	n/a	0
1	Alpha aminoadipate reductase (238030060) [CAY67983.1]	1400	4	0	0	n/a	0	n/a	n/a	0
2a	Cobalamin‐independent methionine synthase (238030843) [CAY68766.1]	768	12	1	0	Sal k 3	630	77.5%	Sal k 3	21
2b	Aconitase (254564667) [XP_002489444.1]	780	14	2	0	n/a	0	n/a	n/a	0
3	Transketolase (238030057) [CAY67980.1]	679	20+	0	0	n/a	0	n/a	n/a	0
4	Glycerol kinase (238034027) [CAY72049.1]	621	20+	0	0	n/a	0	n/a	n/a	0
5a	Catalase A (254569930) [XP_002492075.1]	510	20+	4	0	Pen c 30	214	60%	n/a	0
5b	G6PD (238031000) [CAY68923.1]	504	6	1	0	Bla g 3	10	37%	n/a	0
5c	Hypothetical protein PAS (238031215) [CAY69138.1]	525	20+	8	2 (Cla h 10, Alt a 10)	Cla h 10	442	72.5%	Cla h 10	41
						Alt a 10	442	72.5%	Alt a 10	27
						Lep d 13	1	35.4%		
6	Mitochondrial ALDH (238033249) [CAY71271.1]	501	20+	8	2 (Cla h 10, Alt a 10)	Cla h 10	422	76.2%	Cla h 10	41
						Alt a 10	422	76.2%	Alt a 10	25
7a	Delta‐aminolevulinate dehydratase (238033645) [CAY71667.1]	341	9	0	0	n/a	0	n/a	n/a	0
7b	Mitochondrial alcohol dehydrogenase isozyme III (238031179) [CAY69102.1]	350	12	1	1 (Cand a 1)	Cand a 1	271	85%	Cand a 1	49
7c	Malate dehydrogenase (238034064) [CAY72086.1]	342	18	4	1 (Mala f 4)	Mala f 4	216	70%	Yeast Mala f 4	25
						Pis s 2	7	36.2%		
7d	Putative protein, unknown function (238033788) [CAY71810.1]	328	10	1	0	n/a	0	n/a	n/a	0
8	Triose phosphate isomerase (238032989) [CAY71012.1]	248	10	5	4 (Tri a 31, two isoforms of Der f 25, Cra c 8)	Tri a 31	169	62.5%	Der f 25	17
						Der f 25.0101 (isoform)	169	60.0%	Der f 25	17
						Der f 25.0201 (isoform)	169	60.0%	Cra c 8	11
						Cra c 8	169	57.5%	Tri a 31	17
9a	Hypothetical protein (cyclophilin) PP7435 (328350030) [CCA36430.1]	161	17	7	7 (PPIasef) from fungus: Asp f 27, Der f 29, Mala s 6, Cat r 1, PPIase from birch: Bet v 7, from carrot *Daucus carota*, and from Fungus: Asp f 11)	Mala s 6	82	87.5%	Asp f 27	23
						Asp f 27	82	85%	Der f 29	23
						Cat r 1	82	81.3%	Bet v 7	11
						Der f 29	82	80%	Cat r 1	19
						Asp f 11	82	80%	(Unassigned by IUIS) PPIase from *Dauces carota*	10
						Bet v 7	82	80%	Mala s 6	31
						(Unassigned by IUIS) PPIase from *Dauces carota*	82	78%	Asp f 11	17
9b	Cytosolic superoxide dismutase (238034030) [CAY72052.1]	154	20+	23	23 (isoforms of Ole e 5)	Ole e 5 23 isoforms	75	60 to 55%	n/a	0
10	Mitochondria ATPase inhibitor (238029769) [CAY67692.1]	84	17	4	0	n/a	0	n/a	n/a	0

^a)^ Protein band numbers correspond to the numbers labeled on the stained gel in Figure 1;

^b)^ AAs, amino acids;

^c)^ ID, identity (%);

^d)^ Allergen name in IUIS allergen list unless denoted as unassigned by IUIS;

^e)^ n/a, not available or no answer.

^f)^ PPIase, peptidylprolyl isomerase.

All of the 17 *Pichia pastoris* proteins are enzymes or an enzyme inhibitor (protein #10) that are ubiquitous in nature. Therefore, a search of the NCBI database for sequences related to each of the 17 proteins, using BLASTP without keyword limits identified high scoring alignments with related proteins from many molds and yeasts. In all cases, these included *Saccharomyces cerevisiae* and *Saccharomyces bayanus*, which are commonly used in making wine, bread, and beer and *Saccharomyces boulardii* which is widely used as a probiotic.^37^, ^38^, ^39^ The long history of consumption of organisms producing these close homologs of the 17 *Pichia sp*. proteins with no reports of allergenicity or toxicity offers strong general evidence for their safety in food.

Seven of the 17 *P. pastoris* proteins did not have significant alignments with proteins in the AOL database or among the known allergens or toxins in NCBI, while ten of the 17 had matches that are discussed below (Table 2).

The 1400 AA *P. pastoris* alpha aminoadipate reductase (Protein #1, Table S6, Supporting Information) had no significant matches to proteins in AOL or to known allergens or toxins in NCBI.

**Figure 2:**
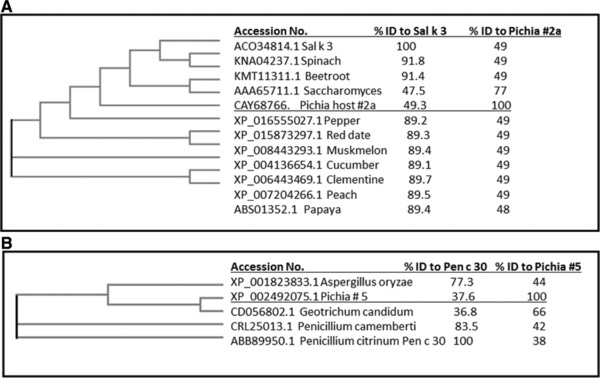
Two phylogenetic trees as examples comparing host *Pichia pastoris* proteins #2a (A) and #5 (B) to sequences of matched allergens from Allergenonline.org, and sequences of proteins from edible plants, and edible fungi *Saccharomyces sp*. and *Aspergillus oryzae*. The edible plant proteins and fungal proteins were identified by BLASTP of the NCBI non‐redundant database and a close homologue of the *Pichia* protein from an edible fungal source.

The 768 AA *P. pastoris* cobalamin‐independent methionine synthase (Protein #2a, Table S7, Supporting Information) had a significant match (77% in the 80‐mer search and one identical eight AA match) to a protein in AOL: pollen allergen Sal k 3, a homologous protein from from Russian thistle (*Salsola kali*, Table 2). However, a phylogenetic tree (www.ebi.ac.uk/Tools/msa/clustalo/) constructed with sequences of *Pichia* host protein #2a, a homologous methionine synthase from *S. cerevisiae*, Sal k 3 and homologues of Sal k 3 in commonly consumed plants. As expected, the phylogenetic tree in **Figure** 2A shows that allergen Sal k 3 has a much closer relationship to homologous proteins from plants to which it has the highest sequence identities. There is no evidence that these homologous proteins (89–91% identity) from plants are allergens or share IgE cross‐reactivity with Sal k 3, thus, it is unlikely that the match of Sal k 3 with the far more distantly related *P. pastoris* protein represents an allergy risk. In addition, the protein from the widely consumed *S. cerevisiae* has high sequence identity (77%) to the host protein #2a and is similarly lower in identity to homologues of Sal k 3 in edible plants.

The 780 AA *P. pastoris* aconitase (Protein #2b, Table S8, Supporting Information) had low identity matches (not statistically significant) to homologous allergenic enzymes of *Aspergillus* sp. and the scabies mite. The low degree of identity suggests the risk of cross‐reactivity is low, and the higher degree of similarity of the *P. pastoris* protein with aconitase from widely consumed *Saccharomyces* species, with no reports of aconitase allergies, suggests that the low identity matches to *Aspergillus* and scabies mite enzymes represent very low risk of allergenicity. Protein #2b had modest identity match to some proteins from toxic organisms, but not to any known toxins themselves. Higher scoring matches to proteins from widely consumed nontoxic yeasts and molds suggests that it is unlikely to pose any risk to humans.

The 679 *P. pastoris* AA transketolase (Protein #3, Table S9, Supporting Information) had no significant alignments to allergens or toxins in either AllergenOnline.org or the NCBI.

The 621 AA *P. pastoris* glycerol kinase (Protein #4, Table S10, Supporting Information) had no significant alignments to any allergens, and only modest identity scores (<42%) with homologous proteins found in bacterial species that have evidence of toxicity to insects or vertebrates. The matched proteins, however, are found in virtually all species and are not known to be toxins to animals (e.g., glycerol kinases).

The 510 AA *P. pastoris* catalase A (Protein #5a, Table S11, Supporting Information) matched the homologous mold allergen Pen c 30 (*Penicillium citrinum*) in the 80 mer AA match with 60% identity to the best 80‐mer match (Figure 2B). However, the homologous protein from the closely related *Penicillium camemberti* is widely consumed in brie and other soft‐ripened cheeses. It is 83% identical to Pen c 30 over its entire length.^40^ While there are two publications reporting allergenicity to *Penicillium camemberti*,^41^, ^42^ there are no reports of food allergy associated with the enzyme. A protein from *Aspergillus oryzae*, which is widely used in Asia as koji in soy sauce and soy paste fermentation^43^ also has a closer identity match to Pen c 30 than that represented by the *P. pastoris* catalase A. These widely consumed soybean‐based foods do not have a history of allergenicity. Instead, they have catalases that have higher identity to Pen c 30 than it is to the *Pichia* protein, suggesting that the *Pichia* protein poses little risk of allergenicity. Finally, *Pichia* catalase is more closely related to catalase from the widely consumed *Saccharomyces* species discussed above (66% identity), with no known history of allergies. Collectively, these results suggest that the risk of allergenic cross‐reactivity of *Pichia* catalase to Pen c 30 is very low.

The 504 AA *Pichia* glucose 6 phosphate dehydrogenase (G6PD, Protein #5b, Table S12, Supporting Information) shared 37% identity with the Bla g 3 protein from *Blattella germanica* (German cockroach). However the G6PDs from all widely consumed fungi (including baker's yeast, *Saccharomyces*, as well as mushrooms and morels) are far more similar to the *Pichia* protein than is Bla g 3. All have sequence similarities to Bla g 3 comparable to that of *Pichia* G6PD, indicating that there is a low risk of allergenic cross‐reactivity between the *Pichia* protein and Bla g 3.

The 525 AA *Pichia* aldehyde dehydrogenase (ALDH, Protein #5c, Table S13, Supporting Information) matched ALDH Cla h 10 (58% identity and 1 × 10^–126^ *E* score), and ALDH Alt a 10 (58% identity and 1 × 10^–124^ *E* score), which are identified as putative mold inhalation allergens based only on low‐level IgE binding.^44^ The sliding 80‐mer window and a number of eight AA identity matches were also significant for these proteins. An additional allergen, Lep d 13, from the storage mite, also yielded significant alignment using the 80‐mer search window, although with a far lower score. ALDH are found in most organisms. A search of the NCBI Protein database without filters using the keyword “allergen” identified numerous proteins with equal or greater similarity to Cla h 10 and Alt a 10, from food organisms commonly consumed as food by humans, including proteins from the commonly consumed yeast species, *Saccharomyces sp*. Moreover, the *Pichia* ALDH matched the ALDH from widely consumed *Saccharomyces* and other yeasts, with no history of ALDH allergies, at much higher similarity scores. We, therefore, conclude that the risk of cross‐reactivity between *Pichia* ALDH and either the mold or mite allergens is very low.

The 501 AA *Pichia* mitochondrial ALDH (Protein #6, Table S14, Supporting Information) was also identified as having significant matches to the Cla h 10 and Alt a 10 proteins with 76% identities in optimal 80 AA alignments, as well as a number of eight AA identity matches and overall FASTA alignments (Table S14, Supporting Information). As noted above, the greater similarity of the matched putative allergenic proteins to commonly consumed proteins with no evident allergenicity demonstrates that these alignments do not suggest a significant risk of food allergy.

The 341 AA *P. pastoris* delta‐aminolevulinate dehydratase (Protein #7a, Table S15, Supporting Information) did not show significant alignments with any known allergens or toxins.

The 350 AA *P. pastoris* mitochondrial alcohol dehydrogenase (Protein #7b, Table S16, Supporting Information) aligned with the homologous Can a 1 from the yeast *Candida albicans* by overall FASTA and 80‐AA alignment, as well as 8‐mer identity match. These proteins are ubiquitous mitochondrial alcohol dehydrogenases, as reflected by the results of a BLASTP search of the NCBI protein database, with similar sequence matches to numerous widely consumed food organisms without known allergenicity cross‐reactivity.

The 342 AA *P. pastoris* malate dehydrogenase (Protein #7c, Table S17, Supporting Information) aligned to a mold allergen Mala f 4 with 45% overall identity and a highest 80‐mer identity of 70%, including 25 identical matches to eight AA segments, indicating clear homology. Both Cand a 1.0101 and Mala f 4.0101 were partially purified and identified only with light IgE binding, leaving doubt as to their relevance for allergenicity.^45^, ^46^, ^47^ Protein #7c also shared a minor identity match to the pea allergen Pis s 2, with highest score of 36.2% in a segment of seven overlapping 80‐AA alignments. A literature search did not reveal cross‐reactivity between Mala f 4 and any *Pichia pastoris* proteins. A BLASTP search of the NCBI database without any keyword filter uncovered higher sequence identities to homologous proteins from a wide variety of sources, including the widely consumed *Saccharomyces* species, suggesting that the matches to Mala f4 and Pis s 2 do not represent a significant risk of allergenic cross‐reactivity.

The 328 AA *P. pastoris* aldo/keto reductase (Protein #7d, Table S18, Supporting Information) aligned with 24.6% identity to juniper allergen Jun o 4, and with only modest sequence identity matches to a few proteins from organisms associated with toxicity. These low scoring alignments suggested a low risk of allergenicity or toxicity. In contrast, the NCBI BLASTP search without keyword limit yielded higher‐scoring identity matches to commonly consumed food species. Thus, there is little evidence of risk of allergic cross‐reactivity from this protein.

The 248 AA triose phosphate isomerase (Protein #8, Table S19, Supporting Information) had four relatively high scoring identity matches (50–53%) over near full‐length alignments to homologous proteins from diverse sources (wheat, house dust mite, and shrimp). Matches were found using full‐length FASTA, 80‐mer alignment and eight AA identity matches with AOL v16. Triose phosphate isomerase is a highly conserved enzyme; a search of the NCBI database using BLASTP without keyword filters yielded higher scoring identity matches to homologous proteins in species that are widely consumed without reported incidences of allergy, including a 71%‐identity alignment to the enzyme from *Saccharomyces*. Thus, it is unlikely that the *Pichia* triose phosphate isomerase is an allergen.

The 161 AA *P. pastoris* cyclophilin (Protein #9a, Table S20, Supporting Information) had significant alignments to a number of cyclophilins that have been identified as targets of IgE from allergic subjects. We found relatively high identity matches, by overall FASTA, sliding 80‐mer, and 8‐AA matches, to putative allergenic proteins from diverse sources, including Asp f 27, Der f 29, Mala s 6, Bet v 7, Asp f 11, and cyclophilins without IUIS‐approved names from *Catharanthus sp*., and carrot. Some cyclophilins were aligned using a BLASTP search of the NCBI database, filtered for “allergen”, “toxin”, or “toxic” identified additional cyclophilins, but the same search, without those filters found better alignments (>70% identity) to cyclophilins from many fungi including a *Saccharomyces* cyclophilin with 74% identity and a very significant *E* score (9 × 10^–88^). In view of the closer similarity of the *P. pastoris* cyclophilin to its *Saccharomyces* homolog than to any known allergens, the matches to allergens are not likely to be indicative of an allergy risk for the *P. pastoris* cyclophilin.

The 154 AA *P. pastoris* cytosolic superoxide dismutase (Protein #9b, Table S21, Supporting Information) aligned with significant scores to the 23 superoxide dismutase isoforms that have been identified from olive pollen as Ole e 5, which was identified as a putative allergen based on IgE binding using sera from a large number of olive‐pollen allergic subjects. A cyclophilin from the widely consumed *Saccharomyces*, was a far better match to Protein #9b, with 79% identity over the entire length of 154 AA. No evidence was found for allergenic cross‐reactivity between molds and olive pollen.

The 84 AA *P. pastoris* protein (Protein #10, Table S22, Supporting Information) did not show significant alignments with any known or suspected allergens or toxins.

Bioinformatics searches for matches to allergens are intended to identify proteins that are already known to be allergens, or proteins that are nearly identical to an allergen from a different source to predict possible cross‐reactivity. As noted by Aalberse,^14^ overall sequence identity alignments of <50% are unlikely to represent IgE cross‐reactivity. The conservative limits of >35% identity in short segments of 80 amino acids are used to identify protein matches that might have any possibility of IgE cross‐reactivity. The 8 AA identity matches have been largely discounted, but are still required by a number of regulatory agencies. If any match is identified to a known allergenic protein that aligns with higher identity than those limits, there is some expectation that serum IgE tests should be performed to identify IgE cross‐reactivity.^12^ However, IgE binding can be of relatively low affinity or often may be restricted to a single epitope, which generally would not trigger a biological response and testing IgE binding in serum samples from a large number of allergic subjects is impractical. As a negative control in the bioinformatics analysis, we compared the proteins of interest to all known sequences in NCBI protein database using BLASTP. In each case, the negative‐control analysis identified proteins expected to be present in commonly consumed human foods, with no known allergenicity, that had far higher sequence‐identity to the LegHb and *Pichia* proteins of interest than any of the matches we found between these proteins and known allergens.

Most of the putative allergens that had significant sequence similarity to the LegHb of the associated *Pichia* proteins were airway allergens from pollen, fungi, or mites. None of those proteins nor any homologues of those proteins (e.g. cyclophilins, aldolase, and alcohol dehydrogenases) have been reported to induce food allergy. Thus, the evidence from bioinformatics analyses, considered in its entirety, suggests little risk of allergenicity or toxicity from either the LegHb or the associated *Pichia* proteins.

Similarly, none of the results from analyses using BLASTP to search across the complete NCBI protein database suggested a significant risk of allergy or toxicity to consumers. Some of the *Pichia* proteins in the LegHb preparation were highly conserved intracellular enzymes with homologues that have been identified as putative allergens from a few organisms. But, in each case, homologues in organisms commonly consumed as human food had higher identity matches, without evidence of shared allergic reactivity.

Every significant sequence alignment between the 17 *Pichia* proteins of interest and proteins annotated with keywords “toxin” and “toxic” correspond to homologous proteins found in toxic organisms; none of these proteins were themselves known or suspected to be toxic. We could find no published evidence suggesting that any proteins with sequences similar to any of the 17 Pichia proteins might be toxic to humans or other mammals.

### 3.4 In Vitro Pepsin Digestibility Results

**Figure 3:**
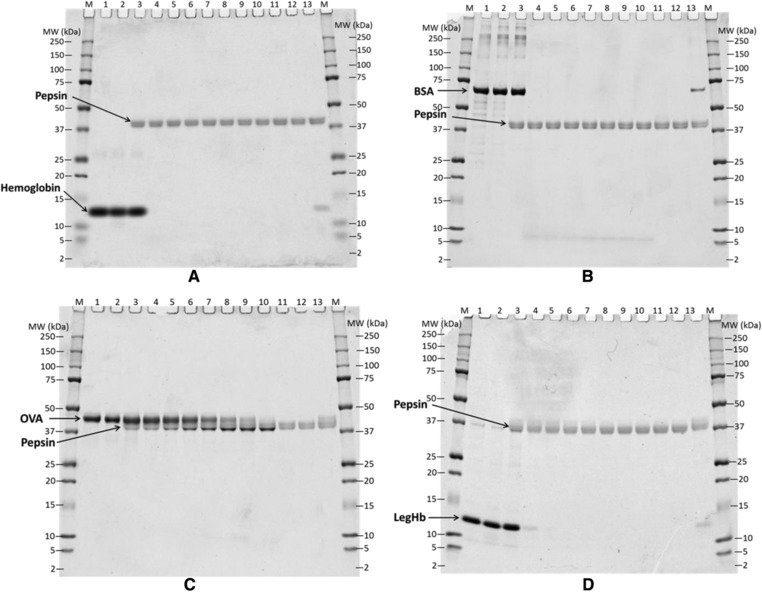
Coomassie Brilliant Blue Stained SDS‐PAGE Gel Showing the Digestion of control samples, Bovine Hb (A), BSA (B) and OVA (C), and LegHb preparation (D) in SGF at the ratio of 10 Units pepsin per μg test protein (pH 2.0). All proteins were loaded 1.47 μg per lane as predigestion concentration. Lane M, molecular weight marker; Lane 1, protein control at 0 min; Lane 2, protein control at 60 min; Lane 3–10, digestion at 0, 0.5, 2, 5, 10, 20, 30, and 60 min; Lane 11, pepsin control at 0 min; Lane 12, pepsin control at 60 min; Lane 13, 10% of undigested protein.

**Figure 4:**
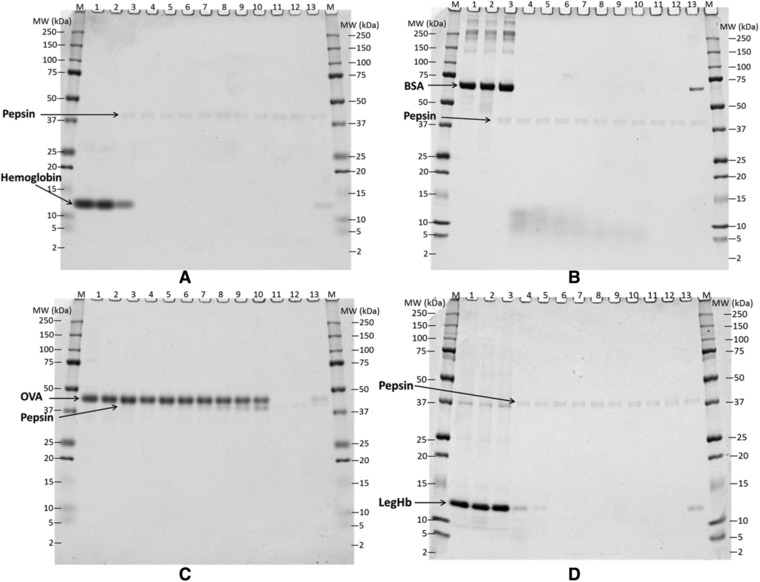
Coomassie Brilliant Blue Stained SDS‐PAGE Gel Showing the Digestion of control samples, Bovine Hb (A), BSA (B) and OVA (C), and LegHb preparation (D) in SGF at the ratio of 1 Unit pepsin per μg test protein (pH 2.0). All proteins were loaded 1.47 μg per lane as pre‐digestion concentration. Lane M, molecular weight marker; Lane 1, protein control at 0 min; Lane 2, protein control at 60 min; Lane 3–10, digestion at 0, 0.5, 2, 5, 10, 20, 30, and 60 min; Lane 11, pepsin control at 0 min; Lane 12, pepsin control at 60 min; Lane 13, 10% of undigested protein.

To assess whether any of the proteins of interest might include digestion‐resistant polypeptides, we carried out in vitro digestion assays at pH 2 in simulated gastric fluid, at pepsin–protein ratios of 10:1 (U μg^–1^) or 1:1 (U μg^–1^), comparing each protein of interest to three control proteins. Under these conditions, two of the control proteins, Hb (**Figure** 3A, **Figure** 4A) and BSA (Figure 3B, and Figure 4B), were digested rapidly, in 30 sec. The third control protein, OVA (Figure 3C and Figure 4C) was considerably more stable, with more than 10% visually stainable full‐length protein band remaining after 30 min digestion with 10 units of pepsin per microgram target protein (Figure 3C) and after 60 min at 1 U μg^–1^ target protein (Figure 4C). At both ratios, digestion of OVA left some residual polypeptides detectable by Coomassie stained SDS‐PAGE as bands with apparent sizes of approximately 38 kDa after 60 min of digestion. The results of these control proteins were consistent with results from previously performed tests,^18^ confirming the reliability of this digestion assay.

Two representative stained gels from digestion of LegHb at pH 2.0 with two ratios of pepsin/protein (Figure 3D and Figure 4D) demonstrated that LegHb was resistant to digestion in acid alone for 60 min (lane 3). However, LegHb was rapidly digested by pepsin in 2 min (lane 5) to below the 10% of the band intensity detected in a control digestion with quenched pepsin (lane 13). Additionally, all *Pichia* protein bands (Figure 1) were digested and no longer visible at time 0.5 min (lane 4 in Figure 3D and Figure 4D), demonstrating they are rapidly digested as well. The results demonstrated that the LegHb protein and the associated *Pichia pastoris* proteins were more than 90% digested within 2 min in SGF plus pepsin at 37 °C, at ratios of either 10 units or 1 unit of pepsin activity per 1 μg of total protein, based on Coomassie Blue staining detection. Thus, there is no added concern of risk based on pepsin stability of this product preparation, according to CODEX^11^ guidelines for the allergenicity assessment.^11^

## 4 Discussion

Bioinformatics comparison of the LegHb protein sequence compared with the protein sequences of known allergens did not identify any significant matches of sufficient concern to warrant serum IgE binding tests for possible cross‐reactivity. The primary soybean allergens are major seed storage proteins, Gly m 5 (three isoforms of beta‐conglycinin) and Gly m 6 (five isoforms of glycinin); and a number of other soybean proteins have been identified as potential allergens (eight in the WHO/IUIS Allergen Nomenclature list; 15 in the OECD list of allergens, some of which have not had sequences determined). These allergens and potential allergens come from the edible bean, whereas LegHb is expressed exclusively in the root nodule. Sequences of all published IgE binding proteins from soybean are included in AllergenOnline.org, which was the primary sequence search database used in this study. No significant sequence similarities were identified, indicating that LegHb is not homologous or significantly similar to any known allergen or IgE reactive protein in soybean. Though soybean is considered a commonly allergenic food source, soybean allergies are prevalent in infants and toddlers through IgE binding to proteins from the beans. Most of those allergic to soybeans become tolerant to soybean ingestion by the age of 10. Allergy to soy is uncommon in teenagers and adults.^48^ Clinical cross‐reactivity among various foods from the legume family in children is rare.^49^ Taken together, these results suggest that soy LegHb is unlikely to pose any risk of food allergy to soy‐allergic or legume‐allergic consumers.

*Pichia pastoris* is commonly used to produce recombinant proteins for cosmetics and pharmaceuticals. Bioinformatics searches with the 17 residual *Pichia* proteins found in soy LegHb preparation identified a few related protein sequences with sufficient similarity to exceed the CODEX suggestion for potential cross‐reactivity. However, the sequence‐related putative allergens we identified in this search were not potent, common allergens, nor were any of them known to be allergenic when orally consumed. As the LegHb protein composes only up to 0.8% in the final plant‐based meat products, the abundance of each Pichia protein is very trace amount. Moreover, comparison of the same *Pichia* proteins with all proteins in the NCBI Protein database found far more significant matches to proteins from commonly consumed fungi, including baker's yeast (*Saccharomyces* species). Impossible Foods’ engineered *Pichia* production strain complies with the Organisation for Economic Co‐operation and Development (OECD) criteria for Good Industrial Large Scale Practice (GILSP) microorganisms.^50^ It also meets the criteria for a safe production microorganism as described by Pariza and Foster,^51^ Pariza and Johnson,^52^ and several expert groups^53^, ^54^, ^55^, ^56^, ^57^ (FAO/WHO).^58^

Although the overall CODEX paradigm for evaluating potential allergenicity has indicated taking a protein from a common source of allergy should trigger serum IgE testing using sera from those with allergy to the source; soybean (seeds) is a common allergenic food source, but root nodules are not. LegHb is not expressed in the bean. Thus, there is no scientific rationale for tests using sera from soybean allergic subjects to determine whether there is IgE binding to LegHb. The other possibility is whether the protein is similar to any known allergen. The AA sequence of LegHb was evaluated by extensive bioinformatics comparison and there was no significant similarity to any allergenic protein. Additionally, the AA sequences of the residual *Pichia* proteins were evaluated by bioinformatics. There were no clear significant identity matches of *Pichia* proteins to known allergens. The LegHb and associated *Pichia* proteins were rapidly digested in simulated gastric fluid (pepsin). While these results do not prove that risks of allergy are nonexistent, they demonstrate a very low likelihood of food allergy, and a lack of need for serum IgE tests or additional biological assays such as skin prick tests, basophil assays or challenges.^11^, ^59^, ^60^, ^61^

A BLASTP search of the NCBI protein database for toxic proteins related by sequence to either LegHb or the associated *Pichia* proteins found no evidence suggesting that any of these proteins pose a risk of toxicity. While a number of organisms with known toxicity (e.g., *Bacillus sp*., *Enterococcus faecalis*, *Streptomyces sp*., and *Clostridium sp*.) contained proteins with sequences similar to those of the *Pichia* proteins of interest, these proteins were ubiquitous and highly conserved across diverse species and are not themselves known or suspected to be toxic. As a further evaluation step, comparison of the sequence‐related proteins from toxin‐producing species to proteins from diverse nontoxic species identified far more closely related proteins from sources known to be safe and nontoxic.

In summary, our analysis found no evidence to suggest that food products containing the LegHb protein and associated minor components from *Pichia pastoris* pose any significant risk of allergy or toxicity to consumers.

**Abbreviations**
AA: amino acid
ALDH: aldehyde dehydrogenase
AOL: AllergenOnline database
FAO: Food and Agricultural Organization
G6PD: glucose 6 phosphate dehydrogenase
Hb: hemoglobin
HOSU: history of safe use
LegHb: leghemoglobin
NCBI: National Center for Biotechnology Information
OVA: ovalbumin
REP: recombinantly expressed protein
SGF: simulated gastric fluid

## Conflict of Interest

R. Z. F is an employee of Impossible Foods, the developer of the food product. R. E. G has received consulting fees for work with Impossible Foods for recommendations for food safety evaluation procedures. The other authors declare that there are no conflicts of interest.

## Supporting information

Supporting Information
