# Supplementary material for: Evaluating Potential Risks of Food Allergy and Toxicity of Soy Leghemoglobin Expressed in Pichia pastoris
Source: Mol Nutr Food Res. 2017 Oct 17;62(1):1700297. doi: 10.1002/mnfr.201700297 (PMC5813221; doi:10.1002/mnfr.201700297)
Supplement: Supplementary file 1 — Supporting Information [file MNFR-62-na-s001.docx]

**Table S1 Amino acid sequences of 17 proteins from *Pichia pastoris* (synon. *Komagataella phaffi*) in the product.** Protein band numbers are designated as in Figure 1. Both GI and Accession numbers are listed as NCBI modified their format during this study and they no longer list GI numbers.

| **Organism** | **Protein, GI, band# and % coverage by LC-MS/MS** | **Native protein sequence** |
| --- | --- | --- |
| *Komagataella phaffii*  (*Pichia pastoris*) | Band #1: Alpha aminoadipate reductase (1400 AA) GI:238030060 Acc: CAY67983.1  32% | MSEENLNYWANILDGPTLSVLPRDYNRPVAGKVIEANKTFDISDILPFLNKANEPSVTQFTAPLAVFAVLVYRLTGDDDIVILT DSPKKQNLPFVVRLQVDPSKSFVDVSKQVGEQYLESLERATPLKDIVTHLKESKQLPNYPPIFRLSFQTAKKVQQLSTLVEGST RDLAIFLENNTSINIYYNSLLYTHNRIAYFSQQFSSFIDEVNKAPETPIGKISLLTEQQSKLLPDPTANLDWSGYRGAIQDIFSDN AEKFPDRTCVVETKSFLNPNSQTRTFTYKQIDQASNIVGNYLVHTGIKRGDVVMIYAYRGVDLMVAVMGVLKAGATFSVID PAYPPARQNVYLQVAKPAGLIVLEKAGVLDQLVEDYIKNELSLVSRISNLKIEADGNVLGGDVDGKDALYDYQQFKTRRTGVL VGPDSNPTLSFTSGSEGIPKGVLGRHFSLAYYFPWMSKTFNLSENDKFTMLSGIAHDPIQRDMFTPLFLGAQLLIPTSDDIGT PGKLAEWMQTYGATVTHLTPAMGQLLSAQATKEIPSLHHAFFVGDILTKRDCLRLQTIAQNVNIINMYGTTETQRAVSYFEI PSRAQDSTFLEVQKDIMPAGKGMHNVQLLVVNRHDRSKTCAIGEVGEIYVRAAGLAEQYRGQPDLNKEKFVPNWFVSPSK WVEEDKKISKDEPWREFYLGPRDRLYRTGDLGRYLPTGDCEVSGRADDQVKIRGFRIELGEIDTHISRHPLIRQNVTLVRRDK DEEPILISYVVPKETPELENFKSSSDDLDDLNDPIVKSLLLYRELIKDLKAHLKKTLASYAIPTIIVPMAKLPLNPNGKVDKPKLPFP DTVQLAAVAQKSSAEVDDSEFTTTELQIKDLWLQVLPNPPASISLEDSFFDLGGHSILATRMIFELRRKLAVDLPLGTIFKHPTV KLFAAEVDRVKNGDEVQFADNKQESTSAGSDEQVVDYFQDAKDLVSSQLLDSYKSRLALSNAELINIFLTGATGFLGSYILKDL LERDLDVQVYAHVRAKDEESGLERLRNTGKVYGIWNEEWTSRIKVVIADLSKDKLGLSGEKYAELANTIDLIIHNGALVHWVY PYSKLRDANVISTINVLNLAASGKPKQFGFVSSTSTLDTEHYITLSDTLTEQGEDGIPESDDLLGSSKGLGTGYGQSKWAAEYII RRAFERGLRGAIIRPGYVTGHSRTGACNTDDFLLRMLKGCAELGKLPNISNTVNMVPVDHVALVVTASSLHPTAEEGHCVV QVTGHPRIRFNEFLNALNDYGYEVKLTDYVEWKRDLERFVVDQSKDSALYPLLHFVLDNLPQDTKAPELDDKNAKDILSGDT RWTGYDGSKGRGVDSAQTGIYIAYLIKTGFLPPPSKEGKKPLPEIEISEESLKLIKEGAGARTSAA |
| *Komagataella phaffii*  (*Pichia pastoris*) | #2a: Cobalamin- independent methionine synthase (768 AA) GI:238030843  Acc: CAY68766.1  77% | MVQSSVLGFPRIGAFRELKKTTEAYWSGKVGKDELFKVGKEIRENNWKLQKAAGVDVIASNDFSYYDQVLDLSLLFNAIPER YTKYELDPIDTLFAMGRGLQRKATDSEKAVDVTALEMVKWFDSNYHYVRPTFSHSTEFKLNGQKPVDEYLEAKKLGIETRPV VVGPVSYLFLGKADKDSLDLEPISLLEKILPVYAELLAKLSAAGATSVQIDEPILVLDLPEKVQAAFKTAYEYLANAKNIPKLVVAS YFGDVRPNLASIKGLPVHGFHFDFVRAPEQFDEVVAALTAEQVLSVGIIDGRNIWKADFSEAVAFVEKAIAALGKDRVIVATS SSLLHTPVDLTNEKKLDSEIKNWFSFATQKLDEVVVVAKAVSGEDVKEALSVNAAAIKSRKDSAITNDADVQKKVDSINEKLSS RAAAFPERLAAQKGKFNLPLFPTTTIGSFPQTKDIRINRNKFTKGEITAEQYDTFIKSEIEKVVRFQEEIGLDVLVHGEPERNDM VQYFGEQLKGFAFTTNGWVQSYGSRYVRPPVVVGDVSRPHAMSVKESVYAQSITKKPMKGMLTGPITVLRWSFPRNDVS QKVQALQLGLALRDEVNDLEAASVEVIQVDEPAIREGLPLRSGQERSDYLKYAAESFRIATSGVKNTTQIHSHFCYSDLDPNHI KALDADVVSIEFSKKDDPNYIQEFSNYPNHIGLGLFDIHSPRIPSKEEFIARIGEILKVYPADKFWVNPDCGLKTRGWEEVRASL TNMVEAAKTYREKYAQN |
| *Komagataella phaffii*  (*Pichia pastoris*) | #2b: Aconitase (780 AA) GI:254564667  Acc: XP_002489444.1  57% | MLSARRVLAKINSRGLATVSGLTKDSLVEMNLLEKGNYINYKQQLDNVNIVKERLGRPLTYAEKLLYGHLDKPHEQDIERGVS YLKLRPDRIACQDATAQMAILQFMSAGMPSVATPTTVHCDHLIQAQKGGAADLERAIRLNKEVYDFLATACAKYNIGFWKP GSGIIHQIVLENYAFPGELLIGTDSHTPNAGGLGQLAIGVGGADAVDVMAGLPWELKAPKIIGVKLTGRMNGWTSPKDIILK LAGITTVKGGTGAIVEYFGDGVDTFSCTGMATICNMGAEIGATTSVFPFNNSMVDFLDATGRSEIGEFAKVFQKEYLSADPG CEYDQVIEIDLNTLEPHINGPFTPDLATPVSKMKEVAVANDWPLEVKVGLIGSCTNSSYEDMTRAASIIEDAASHGVKAKSLT VTPGSEQIRATIARDGQLKTFTDFGGSVLANACGPCIGQWDRQDIKKGDKNTIVSSFNRNFTSRNDGNPATHAFVASPEMV TAYAIAGDLRFNPLTDKLKDKDGNEFLLKDPVGVGLPVRGYDPGENTYQAPPEDRASVEVVISPSSDRLQRLTPFQPWDGK DAERLPILIKSVGKTTTDHISMAGPWLKYRGHLQNISNNYMIGAINAENGEANNVKNHYTGVYSGVPDTAAAYRDNGVKW VVIGGENFGEGSSREHAALEPRYLGGFAIITKSFARIHETNLKKQGLLPLNFTDPAAYDRIQPDDEVDILGLTELAPGKNVTLRV HPADGSPTWETPLSHTYNAEQIEWFKYGSALNNMAAVKASK |
| *Komagataella phaffii*  (*Pichia pastoris*) | #3: Transketolase (679 AA) GI:238030057  Acc: CAY67980.1  63% | MSDLLAINTIRLLAVDTVAKANSGHPGAPMGLAPAAHTLFKQMRFNPRNPAWINRDRFVLSNGHACALLYTMLFLYGYDY TIDDLKSFRQLNSKTPGHPEAELPGVEVTTGPLGQGIANAVGLAIAQAQLAATYNKPNYELFSNYTYAFLGDGCLQEGVAQE AISLAGHLGDGLKLIAFWDDNQISIDGDTNVSFTEDVPAKFRAQGWEVLSVKDGNDDLEGIAAALAKAKTTNKPTLIRLPTII GYGSLQQGTHGVHGSPLKPDDIKQLKKKFGFDPEQNFVVPREVTESYAKHVADNQQVEVEWNKLLTAYTKEYPELGQELH RRLDGKLPENWQKALPTYTVDDKPVASRKLSEIVLTSIEKELPELVGGSADLTGSNLTRWPDAVDFQPKSTGLGDFSGRYFRF GVREHGMGAIINGISAYGANFKAYGGTFLNFVSYASGAVRLSALSGHPIIWVATHDSIGLGEDGPTHQPIETLAHLRALPNL MVWRPADGNETSAAYLRAIESKHTPSIIALTRQNLPQLEHSSIEKAAKGGYTVYPVENPDIILVASGSEVSIAIDGAKKLGTEGV KASVVSIPDFFTFDSQARSYQLSVLPDGVPIMSVEVMSTFGWSKYSHEQFGINRFGISGPGPEIYKFFEFTAEGVADRASKVV QFYKGKELLSPLNKAFESVHA |
| *Komagataella phaffii*  (*Pichia pastoris*) | #4: Glycerol kinase (621 AA) GI:238034027  Acc: CAY72049.1  61% | MGKDYTPLVATIDIGTTSTRAILFDYHGQEVAKHQIEYSTSAQDDIKRKRSQIISSEGISLTVSDDLEVESVDNKAGPTLQFPQP GWVECRPSHILANAVQCLAACLVTMENKNLDRDEKNKYKLISIGVANMRETTVVWSKKTGKPLYNGIVWNDTRNNDIVDE YTAKYSEKEREEMRTLCGCPISTYFSATKFRWLLKHVPEVKQAYDNADGDLMFGTIDSWLIYHLTNEKSHVTDVTNASRTNF MNIETNKYDDRLLKFWDVDTSKVILPEIRSSAEVYGHFKVPHLESIGYVESYLTDDALALLETIEGAPLAGCLGDQSASLVGQL AVRKGDAKCTYGTGAFLLYNTGDQTLISEHGALTTVGYWFPGLDESEDGKHSSKPQYALEGSIAVAGSVVQWLRDNLRLISK AQDVGPLASQVDNSGGVVFVPAFSGLFAPYWDSNSRGTIFGLTQYTSASHIARAALEGVCFQTRAILKAMISDAGASADFLE ESSKATGHNPLSVLAVDGGMSKSDEMMQIQADILGPCVTVRRSINPECTALGAAIAAGFGVPKEDRIWGSLKECTEAILEGN KMYLAAGNTSLDFKATLSDEVRRKEWRLWENAIAKAKGWLKDTA |
| *Komagataella phaffii*  (*Pichia pastoris*) | #5a: Catalase A (510 AA) GI:254569930  Acc: XP_002492075.1  86% | MSQPPKWTTSNGAPVSDVFATERATFDNANHANNAPKVGPLLLQDFQLIDSLAHFDRERIPERVVHAKGAGAFGEFEVTD DISDVCAAKFLDTIGKKTRIFTRFSTVGGEKGSADSARDPRGFSTKFYTEEGNLDLVYNNTPIFFIRDPSKFPHFIHTQKRNPAT NLKDANMFWDYLVNNQESIHQVMYLFSDRGTPASLRKMNGYSGHTYKWYNKKGEWVYVQVHFKSDLGVVNFNNEEAG KLAGEDPDYHTGDLFNAIERGEYPSWTCYIQTMTQEQAAKQPFSVFDLTKVWPHKDFPLRRFGKFTLNENPKNYFAEVEQA AFSPSHTIPSMQPSADPVLQSRLFSYPDTHRHRLGVNYQQIPVNCPVAPVFTPQMRDGSMTVNGNLGSTPNYKSSFCPFST EAQIQTNSHTPEEVLAAHTEKFHWGGILDSKSYDFEQPRALWKVFGKTPGQQRNFCHNVAVHVAAANHEIQDRVFEYFSK VYPEIGDQIRKEVLQLSPRGDSAARL |
| *Komagataella phaffii*  (*Pichia pastoris*) | #5b: Glucose 6 phosphate dehydrogenase (G6PD) (504 AA) GI:238031000 Acc: CAY68923.1  62% | MTDTKAVEFVGHTAIVVFGASGDLAKKKTFPALFGLYREGYLSNKVKIIGYARSKLDDKEFKDRIVGYFKTKNKGDEDKVQEFL KLCSYISAPYDKPDGYEKLNETINEFEKENNVEQSHRLFYLALPPSVFIPVATEVKKYVHPGSKGIARIIVEKPFGHDLQSAEELL NALKPIWKEEELFRIDHYLGKEMVKNLLAFRFGNAFINASWDNRHISCIQISFKEPFGTEGRGGYFDSIGIIRDVIQNHLLQVLT LLTMERPVSNDPEAVRDEKVRILKSISELDLNDVLVGQYGKSEDGKKPAYVDDETVKPGSKCVTFAAIGLHINTERWEGVPIIL RAGKALNEGKVEIRVQYKQSTGFLNDIQRNELVIRVQPNEAMYMKLNSKVPGVSQKTTVTELDLTYKDRYENFYIPEAYESLI RDAMKGDHSNFVRDDELIQSWKIFTPLLYHLEGPDAPAPEIYPYGSRGPASLTKFLQDHDYFFESRDNYQWPVTRPDVLHK M |
| *Komagataella phaffii*  (*Pichia pastoris*) | #5c: Hypothetical protein PAS (525 AA) GI:238031215  Acc: CAY69138.1  77% | MLRTSPATKKALKSQINAFNVAALRFYSSLPLQVPITLPNGKTYNQPTGLFINNEFVPSKQGKTFAVLNPSTEEEITHVYESRED DVELAVAAAQKAFDSTWSTQDPAERGKVLNKLADLIEEHSETLAAIESLDNGKAISSARGDVGLVVAYLKSCAGWADKVFG RVVETGSSHFNYVRREPLGVCGQIIPWNFPLLMWSWKVGPALATGNTVVLKTAESTPLSALYVSQLVKEAGIPAGVHNIVS GFGKITGEAIATHPKIKKVAFTGSTATGRHIMKAAAESNLKKVTLELGGKSPNIVFNDANIKQAVANIILGIYYNSGEVCCAGS RVYVQSGIYDELLAEFKTAAENVKVGNPFDEDTFQGAQTSQQQLEKILGFVERGKKDGATLITGGGRLGDKGYFVQPTIFGD VTPEMEIVKEEIFGPVVTISKFDTIDEVVDLANDSQYGLAAGIHSDDINKVIDVAARIKSGTVWVNTYNDFHQMVPFGGFGQ SGIGREMGVEALENYTQYKAIRVKINHKNE |
| *Komagataella phaffii*  (*Pichia pastoris*) | #6: Mitochondrial aldehyde dehydrogenase (501 AA) GI:238033249 Acc: CAY71271.1  81% | MTFAPPLEFEIDLPNGLKYTQPLGLFINNEFVEGVEGKLLPVINPCDETKITQVWEASAADVDRAVDAAEDAFNNSVWATQ DPLERGKLMNKLADLIDRDFNILAGIESIDNGKAYTSAQGDVTLAVNYIRSCAGWADKILGNVVDSGNTHLNLVKREPLGVV GQIIPWNFPLLMLAWKLGPALATGNTVVLKTAESTPLSGLYVAKLIKEAGFPPGVVNILSGFGNPAGAAIAAHPRIKKIAFTGS TATGRKIMEAAAKSNLKKVTLELGGKSPNIVFEDADIQKTIHNIILGIFFNSGEVCCAGSRVYIQDTVYEEVLEAFKKETDNVKV GGPFEEGVFQGPQTSELQLNRILSYIKHGKDEGARVITGGSRYRNRGYYIKPTIFADVTEDMKIVKEEIFGPVVTITKFSTVDEV VGYANNTNYGLAAGIHTNNLNKAIDVASRIKAGVVWINTYNDFHHMVPFGGYGESGIGRELGAEALDNYTQAKAIRIAYTP EHK |
| *Komagataella phaffii*  (*Pichia pastoris*) | #7a: Delta- aminolevulinate dehydratase (341 AA) GI:238033645  Acc: CAY71667.1  73% | MVHKAEYLDDHPTQISSILSGGYNHPLLREWQHERQLNKNMFIFPLFVTDRPDEEELIPSLPNIKRFGVNKLIPYVGGLVSKGL RAVILFGVPLKPGVKDEEGTAADDPEGPVIQAIKHLRKNFPDLYIITDVCLCEYTSHGHCGILYEDGTINRELSVRRIAAVAVKY AQAGANSVAPSDMTDGRIRDIKEGLLSAGLAHKTFVMSYAAKFSGNLYGPFRDAAGSCPSQGDRKCYQLPSGGKGLAHRA LIRDMNEGTDGIIVKPSTFYLDIVADAYQLCKDYPICCYQVSGEYAMLHAAAEKNIVDLKSIAFEAHQGFLRAGARLIISYFTPE FLEWLSE |
| *Komagataella phaffii*  (*Pichia pastoris*) | #7b: Mitochondrial alcohol dehydrogenase isozyme III (350 AA) GI: 238031179  Acc: CAY69102.1  84% | MSPTIPTTQKAVIFETNGGPLEYKDIPVPKPKSNELLINVKYSGVCHTDLHAWKGDWPLDNKLPLVGGHEGAGVVVAYGEN VTGWEIGDYAGIKWLNGSCLNCEYCIQGAESSCAKADLSGFTHDGSFQQYATADATQAARIPKEADLAEVAPILCAGITVYK ALKTADLRIGQWVAISGAGGGLGSLAVQYAKALGLRVLGIDGGADKGEFVKSLGAEVFVDFTKTKDVVAEVQKLTNGGPHG VINVSVSPHAINQSVQYVRTLGKVVLVGLPSGAVVNSDVFWHVLKSIEIKGSYVGNREDSAEAIDLFTRGLVKAPIKIIGLSELA KVYEQMEAGAIIGRYVVDTSK |
| *Komagataella phaffii*  (*Pichia pastoris*) | #7c: Malate dehydrogenase (342 AA)  GI: 238034064 Acc: CAY72086.1  77% | MVKVTVCGAAGGIGQPLSLMFKLNPYVTTLALYDVVNVPGVGKDLSHIDTDTKLESYLPENDGLEKALTGSDLVIIPAGVPRK PGMTRDDLFAINAGIIRDLANGIAQFAPSAFVLVISNPVNSTVPIVAEILKKNNVFNPQKLFGVTTLDCVRANTFVAELSKDKE ASAFDTRVLGGHSGETIVPVFSQSAPEVYKELSDEQKAALVHRVQFGGDEVVKAKNGAGSATLSMAYAGYKLGHALLAAIN DTPNIIESTFVYLKDSKIKGAAEAFKYINEKLKDSDSSDVDFFALPVVLSSNGIEEIKWDILEKVDAKETELLKIATGQLSKNIAKG TAFIAGN |
| *Komagataella phaffii*  (*Pichia pastoris*) | #7d: Putative protein, unknown function (328 AA) GI:238033788  Acc: CAY71810.1  77% | MVVAIEGGTGLGLMNLTWKPTPTPIDDAIETIRYAVEEAGVRYLNGGEFYNFPLDSNLNLQYIQEFAKRYPELYKKVSLSVKG AVSLVDVSPDSSPENLEKSISNITKHLPNNFLPIFEPARIDKRYSIEETIKNLSKFVEDGRIGGISLSEVGADTIRRAAKVAPIACVE VEFSLLTRDILHNGVLAACEDLNIPIIAYSPLGRGFLTGTINSKADIPEGDIRLSLERFNDDEVIEHNLKLVHGLKKIADKKGVTLA QLSLAWLRKFGDKHVKVLPIPSCSSPRRVAENTKEISLTDSEFQEITDFAESVPIKGGRYNKASEAVLNG |
| *Komagataella phaffii*  (*Pichia pastoris*) | #8: Triose phosphate  isomerase (248 AA) GI:238032989  Acc: CAY71012.1  73% | MARTFFVGGNFKMNGSKKSIHEIIERLNNTKLPENVEVVIAPPAPYLQQAVTENKQKTVYVSAQNSFDKASGAYTGEVSVEA LKDLGVPYVILGHSERRTINKEDDAFIASKTKFALDQGLKVILCIGETLEEKQANITLDVVKRQLQAVVDVVSDWTNIVVAYEP VWAIGTGLAATPSDAQDVHKQIRDFLATVIGKDQAEKVRILYGGSVNGKNAVEFRDKADVDGFLVGGASLKPEFVDIINSRN |
| *Komagataella phaffii*  (*Pichia pastoris*) | #9a: Hypothetical protein (cyclophilin) PP7435 (161 AA) GI:328350030  Acc: CCA36430.1  66% | MTKTFFDVSSNDQPLGRIVFELYDDVPKTIENFRALCTGEKGYGYKDSIFHRVIPQFMLQGGDFTKFNGTGGKSIYGEKFADE NFIHKHTKPGLLSMANAGPNTNGSQFFITTVPCPWLDGKHVVFGEVVDGLDVVSKIETLGSSSGATKTQLKITNSGEL |
| *Komagataella phaffii*  (*Pichia pastoris*) | #9b: Cytosolic superoxide dismutase (154 AA) GI:238034030 Acc: CAY72052.1  94% | MVKAVAVLRGDSTVGGTVVFEQSSESSPTTITYDIKGNSPNAERGFHIHQFGDNTNGCTSAGPHFNPFGKTHGAPTDEARH VGDLGNVKTDAEGVAKGVITDNQVKLIGETSILGRTVVIHDGTDDLGKGGHADSLKTGNAGGRPACGVIGLAA |
| *Komagataella phaffii*  (*Pichia pastoris*) | #10: Mitochondria ATPase inhibitor (84 AA) GI:238029769  Acc: CAY67692.1  32% | MFQRTTATLVRQNKAIARFYSEGSTGAPRSDGSGDAFTKREKAQEDFYIKKHQAEQLAKLREQLKNQKEHLQNLEKEINNIK EK |

# Table S2 Literature search results for soybean (*Glycine max*) leghemoglobin. The primary and most useful search was with PubMed, but additional searches were performed using Web of Science, Scopus and AGRICOLA. The abstracts and in some cases, whole papers were reviewed to understand relevance.

| ***Glycine max***  **only** | ***Glycine max***  **AND**  **leghemoglobin** | ***Glycine max* AND allergen/allergy** | ***Glycine max* AND toxin/toxic** |
| --- | --- | --- | --- |
| **PubMed search results** | | | |
| 21,936 articles | 201 articles  When allergen was added to the search criteria, no reference came up. | 381 articles for allergen and 665 articles for allergy.  Further searches with leghemoglobin added to either search criteria yielded no results.  One article was cited when searched with Soy AND allergy AND heme but was not relevant to the topic. | 419 articles found for toxin and 342 articles found for toxic.  An additional criterion of either leghemoglobin or heme was added to the search criteria with *Glycine max* AND toxic yielded 1 and 8 results, respectively. None were relevant to the topic. |
| **Web of Science Core Collection search results** | | | |
| 19,230 articles | 90 articles  When allergen was added to the search criteria, no reference came up. | 103 articlesnd for allergen and 35 articles for allergy.  Further searches with leghemoglobin added to either search criteria yielded no results. | 67 articles found for toxin and 276 articles found for toxic.  An additional criterion of either leghemoglobin or heme was added to the search criteria with *Glycine max* AND toxic yielded 2 and 2 results, respectively. None were relevant to the topic. |
| **Scopus search results** | | | |
| 48,222 articles | 143 articles  When allergen was added to the search criteria, no reference came up. | 319 articles for allergen and 283 articles for allergy.  Further searches with leghemoglobin added to either search criteria yielded no results. | 288 articles found for toxin and 679 articles found for toxic.  An additional criterion of either leghemoglobin or heme was added to the search criteria with *Glycine max* AND toxic yielded 3 and 3 results, respectively. None were relevant to the topic. |
| **National Agricultural Library Catalog (AGRICOLA) search results** | | | |
| Over 10,000 articles | 123 articles  When allergen was added to the search criteria, no reference came up. | 31 articles for allergen and 6 articles for allergy. Further searches with leghemoglobin added to either search criteria yielded no results. | 26 articles found for toxin and 135 articles found for toxic.  An additional criterion of either leghemoglobin or heme was added to the search criteria with *Glycine max* AND toxic yielded 2 and 1 results, respectively. None were relevant to the topic. |

**Table S3 Literature search results for the recombinant yeast host *Pichia pastoris* (synonym *Komagataella phaffii*).** Searches with PubMed were most productive. Additional searches were performed using Web of Science, Scopus and AGRICOLA. The abstracts and in some cases whole papers were reviewed to understand the relevance.

| ***Pichia pastoris* or *Komagataella phaffii*** | ***Pichia pastoris***  **AND allergen** | ***Pichia pastoris***  **AND toxin** | **Individual endogenous (17) *Pichia pastoris* proteins *Pichia pastoris* AND individual protein name** |
| --- | --- | --- | --- |
| **PubMed search results** | | | |
| 5,243 articles for *Pichia pastoris*.  6 articles for *Komagataella phaffii*. None of the publications on *K. phaffii* were related to allergens or toxins of the organism. | 128 articles, most relate to expression of heterologous genes and proteins.  5 articles when NOT recombinant was added. | 185 articles  26 articles when NOT recombinant was added.  Carefully reading all the abstracts demonstrated that only one is relevant to topics, which demonstrated that no toxins active against pathogens or other yeasts were identified in *Pichia pastoris* [1]. | Searches with the following individual identified proteins did NOT identify any publications that described allergenicity or toxicity.  Alpha-aminoadipate reductase: 0 article  Cobalamin-independent methionine synthase: 0 articles  Aconitase: 2 articles  Transketolase: 5 articles  Glycerol kinase: 5 articles  Catalase A: 21 articles, 0 article when AND Allerg* added  GAPDH: 1 article  Hypothetical protein PAS: 0 article  Mitochondrial aldehyde dehydrogenase: 1 article  Delta-aminovulinate dehydrogenase: 1 article  Mitochondrial alcohol dehydrogenase: 2 articles  Malate dehydrogenase: 1 article  Unknown function (possibly pyridoxal reductase: 0 Triosphosphate isomerase: 0  Cyclophilin: 0  Cytosolic superoxide dismutase: 3 articles  Mitochondria ATPase inhibitor: 0 |
| **Web of Science Core Collection search results** | | | |
| 8,399 articles for *Pichia pastoris*.  18 articles for *Komagataella phaffii*. None of the publications on *K. phaffii* were related to allergens or toxins of the organism. | 188 articles, most relate to expression of heterologous genes and proteins.  36 articles when NOT recombinant was added. | 172 articles  55 articles when NOT recombinant was added.  The same paper was identified [1]*.* | Searches with the following individual identified proteins did NOT identify any publications that described allergenicity or toxicity.  Alpha-aminoadipate reductase: 0 article  Cobalamin-independent methionine synthase: 0 article  Aconitase: 1 articles  Transketolase: 3 articles  Glycerol kinase: 10 articles  Catalase A: 49 articles, 0 article when AND Allerg* added  GAPDH: 8 articles  Hypothetical protein PAS: 0 article  Mitochondrial aldehyde dehydrogenase: 0 article  Delta-aminovulinate dehydrogenase: 0 article  Mitochondrial alcohol dehydrogenase: 0 article  Malate dehydrogenase: 0 article  Unknown function (possibly pyridoxal reductase): 0 article Triosphosphate isomerase: 0 article  Cyclophilin: 0 article  Cytosolic superoxide dismutase: 0 article  Mitochondria ATPase inhibitor: 0 article |
| **Scopus search results** | | | |
| 7,378 articles for *Pichia pastoris*.  20 articles for *Komagataella phaffii*. None of the publications on *K. phaffii* were related to allergens or toxins of the organism. | 137 articles, most relate to expression of heterologous genes and proteins.  42 articles when NOT recombinant was added. | 121 articles  37 articles when NOT recombinant was added.  One paper [2] investigated *Tetrapisispora phaffii* killer toxin Kpkt which is heterologously produced in *Phicia pastoris*. It is not an endogenous protein from *Pichia*. Besides, Kpkt is known to be active to spoilage yeast in wine-making [3]. Thus, Scopus search failed to find any evidence of toxin existing in *Pichia*. | Searches with the following individual identified proteins did NOT identify any publications that described allergenicity or toxicity.  Alpha-aminoadipate reductase: 0 article  Cobalamin-independent methionine synthase: 0 article  Aconitase: 0 article  Transketolase: 7 articles  Glycerol kinase: 15 articles  Catalase A: 48 articles, 0 article when AND Allerg* added  GAPDH: 9 articles  Hypothetical protein PAS: 1 article  Mitochondrial aldehyde dehydrogenase: 1 article  Delta-aminovulinate dehydrogenase: 0 article  Mitochondrial alcohol dehydrogenase: 5 articles  Malate dehydrogenase: 6 articles  Unknown function (possibly pyridoxal reductase): 0 article Triosphosphate isomerase: 0 article  Cyclophilin: 2 articles  Cytosolic superoxide dismutase: 3 articles  Mitochondria ATPase inhibitor: 0 article |
| **National Agricultural Library Catalog (AGRICOLA) search results** | | | |
| 1,971 articles for *Pichia pastoris*.  7 articles for *Komagataella phaffii*. None of the publications on *K. phaffii* were related to allergens or toxins of the organism. | 13 articles, most relate to expression of heterologous genes and proteins.  5 articles when NOT recombinant was added. | 24 articles  8 articles when NOT recombinant was added. | Searches with the following individual identified proteins did NOT identify any publications that described allergenicity or toxicity.  Alpha-aminoadipate reductase: 0 article  Cobalamin-independent methionine synthase: 0 article  Aconitase: 0 article  Transketolase: 0 article  Glycerol kinase: 2 articles  Catalase A: 7 articles  GAPDH: 4 articles  Hypothetical protein PAS: 0 article  Mitochondrial aldehyde dehydrogenase: 0 article  Delta-aminovulinate dehydrogenase: 0 article  Mitochondrial alcohol dehydrogenase: 1 article  Malate dehydrogenase: 0 article  Unknown function (possibly pyridoxal reductase): 0 article Triosphosphate isomerase: 0 article  Cyclophilin: 0 article  Cytosolic superoxide dismutase: 3 articles  Mitochondria ATPase inhibitor: 0 article |

# Table S4 Overall FASTA3 search of AllergenOnline version 2016 (AOL v16) with the leghemoglobin. Summary results from FASTA3 full-length search of AllergenOnline.org database with the AA sequence of LegHb. Results only identified modest sequence matches to the occupational allergen, hemoglobin from larvae of the *Chironomus thummi*. that are sometimes called blood worms due to high hemoglobin content.

| **Quary Protein** | **Sequence GI# [Acc#]** | **Organism** | **Description** | **Length aa** | ***E* score** | **Identity (%)** | **aa Alignment length** |
| --- | --- | --- | --- | --- | --- | --- | --- |
| leghemoglobin (LegHb) GI:126241  *Glycine max* | 56405052 [P84296.1] | *Chironomus thummi thummi* | Globin  (Insect hemoglobin) | 161 | 0.00024 | 26.2 | 145 |
|  | 121244 [P12548.1] | *Chironomus thummi thummi* | Globin | 161 | 0.00043 | 25.9 | 147 |
|  | 56405054 [P84298.1] | *Chironomus thummi thummi* | Globin | 161 | 0.0009 | 25.0 | 144 |
|  | 121248 [P12549.1] | *Chironomus thummi thummi* | Globin | 161 | 0.0012 | 25.0 | 144 |
|  | 121249 [P12550.1] | *Chironomus thummi thummi* | Globin | 162 | 0.0012 | 25.2 | 151 |
|  | 2506460 [P02221.2] | *Chironomus thummi thummi* | Globin | 158 | 0.0039 | 24.2 | 149 |
|  | 1707908 [P02222.2] | *Chironomus thummi thummi* | Globin | 160 | 0.0053 | 25.2 | 139 |
|  | 121259 [P02228.1] | *Chironomus thummi thummi* | Globin | 151 | 0.0068 | 26.9 | 119 |
|  | 121237 [P02227.1] | *Chironomus thummi thummi* | Globin | 151 | 0.019 | 26.3 | 137 |
|  | 2506461 [P02224.2] | *Chironomus thummi thummi* | Globin | 162 | 0.049 | 24.0 | 150 |
|  | 1707911 [P02223.2] | *Chironomus thummi thummi* | Globin | 161 | 0.057 | 22.9 | 140 |
|  | 121256 [P02231.1] | *Chironomus thummi thummi* | Globin | 151 | 0.32 | 22.1 | 149 |

**Table S5 BLASTP of NCBI Entrez with leghemoglobin using Entrez query keyword “allergen”, “allergy”, “toxic” and “toxin”.** BLAST with keyword “allergy” gave a list of 15 protein sequences on 27^th^ February, 2016 and the top 2 were shown in the table. All are poorly scoring identity –sequence length matches with large *E* scores, suggesting low level homology.

| **Keyword** | **BLAST results** | | | | | | |
| --- | --- | --- | --- | --- | --- | --- | --- |
|  | **Sequence Accession #** | **Organism** | **Description** | **Length aa** | ***E***  **score** | **Identity (%)** | **Query Cover (%)** |
| **allergen** | No matches found | | | | | | |
| **allergy** | EOQ89444.1 | *Leptospira yanagawae serovar Saopaulo* | adenylate/guanylate cyclase catalytic domain protein | 440 | 7e-05 | 29 | 93 |
|  | EJO56091.1 | *Burkholderia multivorans* | nitric oxide dioxygenase | 403 | 0.002 | 43 | 31 |
| **toxic** | No matches found | | | | | | |
| **toxin** | BAO69646.1 | *Bordetella bronchiseptica* | nitric oxide dioxygenase | 402 | 0.027 | 35 | 31 |
|  | ALX21654.1 | *Bordetella pertussis* | dihydropteridine reductase | 402 | 0.028 | 35 | 31 |

**Table S6 Bioinformatics Summary Results of Protein Band #1.** The 1400 AA protein, Alpha aminoadipate reductase, GI: 238030060 was compared with AOL v 16 and with NCBI Protein on 17^th^ May, 2016.

| **AOL full FASTA3** | No match to any protein with an *E* score of < 1 was found. Four low identity short segment matches with *E* scores between 1 and 10. The proteins with short-low identity matches included icarapin from honey bee, ovotransferrin from chicken egg and Ani s 7 from *Anisakis simplex*. |
| --- | --- |
| **AOL 80mer FASTA3** | No match to any protein with identity of >35% over any 80 AA segment. |
| **AOL Exact 8 AA** | No exact 8mer match with any protein. |
| **NCBI BLASTP**  **Allergen** | Two low scoring identity matches to non-homologous, hypothetical proteins with *E* scores >1 e-05, and alignments of < 25% of the length and < 30% identity. |
| **NCBI BLASTP**  **Toxin or Toxic** | A number of low scoring identity matches to homologous proteins (small *E* scores) were found that are proteins from organisms that are classified as toxic to insects (e.g. *Saccharomyces*), but no significant alignment to an obvious toxin or toxic proteins. |
| **NCBI BLASTP**  **No keyword** | Many matches of high scoring identity with protein homologous proteins from other yeasts and molds. The highest scoring match to a *Saccharomyces sp.* protein has an *E* score = 0.0, and 60% identity in a 1418 AA alignment. |
| **Conclusion:** There is a very low risk of Allergy or toxicity identified for protein #1 based on lack of sequence matches to any known allergens. Therefore, risks from consumption of this protein appear to be minimal. | |

**Table S7 Bioinformatics Summary Results of Protein Band #2a.** The 768 AA Cobalamin-independent methionine synthase protein, GI: 238030843 was compared with AOL v16 and with NCBI Protein on 17^th^ May, 2016.

| **AOL full FASTA3** | One significant match was identified to Sal k 3 from Russian thistle (*Salsola kali*). Sal k 3 was first identified as a pollen allergen in 2011 [4]. While it is not clear how important Sal k 3 is an airway allergen, there hasn’t been any publication of allergy upon digestion, and no cross-reactivity between Sal k 3 and any food allergen has been reported. In addition, a BLASTP with Sal k 3 revealed highly homologous proteins from wide food sources that are not known as allergens with much higher identities to Sal k 3 than found with band #2a (Figure 2a). The lack of reported cross-reactivity between Sal k 3 and other common proteins and sources indicate it is unlikely that protein #2a will be a cross-reactive issue. |
| --- | --- |
| **AOL 80mer FASTA3** | The only protein with an 80mer match was Sal k 3, with 77.5% identity as the highest alignment. |
| **AOL Exact 8 AA** | The only protein with an exact 8mer match is Sal k 3. |
| **NCBI BLASTP**  **Allergen** | Two low scoring identity matches to non-homologous, hypothetical proteins with *E* scores >1 e-05, and alignments of < 25% of the length and < 30% identity. |
| **NCBI BLASTP**  **Toxin or Toxic** | A number of low scoring identity matches to homologous proteins (small *E* scores) that are proteins from organisms that are classified as toxic to insects (e.g. *Bacillus thuringiensis*), but no significant alignment to an obvious toxin or toxic proteins. |
| **NCBI BLASTP**  **No keyword** | Many matches of high scoring identity with homologous proteins from other yeasts and molds. The highest scoring match to a *Saccharomyces sp*. protein has an *E* score = 0.0, and 77% identity in a 765 AA alignment. |
| **Conclusion:** There is a minimal risk of allergy based from the modest alignment with Sal k 3. There was no significant match to a toxin, so the likely risk of toxicity is extremely low. Therefore, risks from consumption of this protein appear to be minimal. | |

**Table S8 Bioinformatics Summary Results of Protein Band #2b.** The 780 AA Aconitase protein, GI:254564667 was compared with AOL v16 and with NCBI Protein on 17^th^ May, 2016.

| **AOL full FASTA3** | There were two alignments with *E* scores between 0.1 and 1 (*Aspergillus sp.* endo-chitosane 238 AA, 31% identity over 80 AA; *Sarcoptes scabiei* inactive cysteine protease, 340 AA, 25% identity over 139 AA), and 10 alignments with *E* scores between 4 and 10, various proteases with low identity scores (24% to 37% identity). |
| --- | --- |
| **AOL 80mer FASTA3** | No matches of >35% identity over 80 AA with any protein in AOL v16. |
| **AOL Exact 8 AA** | No exact 8 AA match with any protein in AOL v16. |
| **NCBI BLASTP**  **Allergen** | One very low scoring alignment with an alkyl hydroperoxide annotated as a “Mal allergen” (no reference) from an Antarctic coastal bacteria, genomic sequence. The *E* score was 0.86, 35% identity over 72 AA and three other irrelevant lower identity alignments. |
| **NCBI BLASTP**  **Toxin or Toxic** | A number of statistically significant alignments with *E* scores smaller than 1e-50, one with 0.0 were found. However most of the alignments were modest identities of 50% or less, indicating homology. Most of the source organisms were toxic, but no obvious reference to toxicity of the proteins. There was one higher scoring alignment of 66% with *E* score 0.0, to a bovine homologue of the aconitase enzyme (GI: 157831069, bovine 4-hydroxy-trans-aconitate) can bind to a “toxic” substrate, fluorocitrate. The protein itself is not toxic. And the fluorocitrate and fluoroacetate inhibits the enzyme, causing toxicity. The LegHb and plant based meats do not contain fluoroacetate or flurocitrate and the aconitase protein is not a functional part of food. |
| **NCBI BLASTP**  **No keyword** | Many matches of high scoring identity with protein homologous proteins from other yeasts and molds. The highest scoring match to a *Saccharomyces sp*. protein has an *E* score = 0.0, and 81% identity in a 669 AA alignment. |
| **Conclusion:** There is a very little risk of allergy based on the modest alignment with endo-chitosane and inactive protease. There were moderate alignments to proteins from toxic organisms, but only as common homologues. Thus, the likely risk of toxicity is extremely low. Therefore, risks from consumption of this protein appear to be minimal. | |

**Table S9 Bioinformatics Summary Results of Protein Band #3.** The 679 AA Transketolase protein, GI:238030057 was compared with AOL v16 and with NCBI Protein on 17^th^ May, 2016.

| **AOL full FASTA3** | No matches to proteins in AOL v16 with an *E* score <1. There were >20 matches with *E* score between 1 and 10 with low identities of <35% and they were generally short, 49 to 134 AA. These matches were not significant. |
| --- | --- |
| **AOL 80mer FASTA3** | No identity match of >35% over any segment of 80 amino acids compared to any protein. |
| **AOL Exact 8 AA** | No exact 8 AA matches with any protein. |
| **NCBI BLASTP**  **Allergen** | No significant alignment with any protein in NCBI with using the keyword allergen. |
| **NCBI BLASTP**  **Toxin or Toxic** | Many small *E* score identity matches of just under 50% identity to transketolases of bacteria (e.g. *Bacillus cereus*) or cyanobacteria that are known to be toxic organisms. These are homologues of the enzyme involved in sugar catabolism from photosynthesis. No references were obvious showing any toxic effects of these enzymes on consumers. |
| **NCBI BLASTP**  **No keyword** | Many matches of high scoring identity with protein homologous proteins from other yeasts and molds. A transketolase from the commonly used food yeast, *Saccharomyces cerevisiae* has an *E* score of 0 and 70% overall identity to the transketolase of *P. pastoris*. |
| **Conclusion:** There is no identified risk of allergy or toxicity from the transketolase protein. The higher scoring alignment with transketolase of *Saccharomyces cerevisiae* provides additional assurance of safety. Therefore, risks from consumption of this protein appear to be minimal. | |

**Table S10 Bioinformatics Summary Results of Protein Band #4.** The 621 AA Glycerol kinase protein, GI:238034027 was compared with AOL v16 and with NCBI Protein on 17^th^ May, 2016.

| **AOL full FASTA3** | No matches to proteins in AOL v16 with an *E* score <1. There were >20 matches with *E* score between 1 and 10 with low identities of <35% and they were generally short, 49 to 134 AA. These matches were not significant. |
| --- | --- |
| **AOL 80mer FASTA3** | No matches of >35% identity over 80 AA were found compared to any protein in AOL v16. |
| **AOL Exact 8 AA** | No exact 8 AA matches were identified to any protein in AOL v16. |
| **NCBI BLASTP**  **Allergen** | No significant alignments were identified. |
| **NCBI BLASTP**  **Toxin or Toxic** | A number of low scoring identity matches to homologous proteins (small *E* scores e.g. 6e-118) that are proteins from organisms that are classified as toxic to insects (e.g. *Bacillus thuringiensis*), or mammals (e.g. *Mycobacterium tuberculosis*), with up to 41% identity matches and many gaps over 550 AA, but no significant alignment to an obvious toxin or toxic proteins. |
| **NCBI BLASTP**  **No keyword** | Many matches of high scoring identity with protein homologous proteins from other yeasts and molds were found. The highest scoring alignment with a *Saccharomyces sp.* was with an *E* score of 0.0 and identity of 53% over 664 AA. |
| **Conclusion:** There does not seem to be a risk of allergy based on the lack of alignment to allergens. There are modest alignments to proteins that have not been demonstrated to be toxins, but are from organisms that are known to be toxic. However, with higher identities to proteins from other yeasts, including *Saccharomyces sp*., it is very unlikely that this protein is a toxin. Therefore, risks from consumption of this protein appear to be minimal. | |

**Table S11 Bioinformatics Summary Results of Protein Band #5a.** The 510 AA Catalase A protein, GI:254569930 was compared with AOL v16 and with NCBI Protein on 17^th^ May, 2016.

| **AOL full FASTA3** | One highly significant match with 2.6 e-058 but only 37% identity over 475 AA was identified to a putative allergen Pen c 30, a catalase of *Penicillium citrinum*, identified as an allergen based on IgE binding to mold allergic subjects in Taiwan [6]. There were two Asn-linked glycans on the natural *Penicillium citrinum* protein and the deglycosylated form had reduced binding. There was no published allergic reaction to ingestion of Pen c 30. *Penicillium camemberti* is widely consumed with brie and other soft-ripened cheeses [7], and *Aspergillus oryzae* is widely used in Asia as koji in soy sauce and soy paste fermentation [8], both of which have catalase that are closer to the allergenic ones than is the Pichia protein (Figure 2b). Therefore, cross-reactivity between #5a and Pen c 30 is highly unlikely. |
| --- | --- |
| **AOL 80mer FASTA3** | The only alignment with >35% identity over 80 AA was to Pen c 30 and the best aligned 80 AA segment was 60% identity, indicating restricted conservation of sequence to a subsection of the protein. |
| **AOL Exact 8 AA** | No exact 8 AA matches were identified to any protein, not even to Pen c 30. |
| **NCBI BLASTP**  **Allergen** | Only the single alignment of Pen c 30, with an *E* score of 9e-93 and 38% identity over 424 AA. |
| **NCBI BLASTP**  **Toxin or Toxic** | Many significant (very small *E* score) alignments were identified (e.g. 5e-149 for toxin, 0.0 for toxic) to protein homologues from organisms noted for toxins or toxicity (e.g. *Bacillus sp*., *Enterococcus faecalis*, *Streptomyces sp.*, *Clostridium sp*.). The alignments were approximately 50% for proteins identified with “toxin”, and 71% as the highest for those identified with “toxic” organisms (*Candida boidinii*). The enzyme is common to all organisms living in oxygen containing environments as it detoxifies hydrogen peroxides by converting them to H2O and O2. It is not toxic to mammalian cells. No published evidence was found that catalase A is a toxin. |
| **NCBI BLASTP**  **No keyword** | Many high scoring identity matches were found with homologous proteins from other yeasts and molds. The full-length of #5a represents a conserved catalase A common to most yeast and fungi. The highest scoring alignment with a *Saccharomyces sp*. was with an *E* score of 0.0 and identity of 66% over 494 AA. |
| **Conclusion:** There is a minimal risk of allergy based on a relatively poor alignment to the allergen Pen c 30. There are much higher alignments to other proteins including the catalase A proteins of a variety of yeasts and molds including from *Saccharomyces sp.* There are modest alignments to proteins that have not been demonstrated to be toxins, but are from organisms that are known to be toxic. Therefore, risks from consumption of this protein appear to be minimal. | |

**Table S12 Bioinformatics Summary Results of Protein Band #5b.** The 504 AA glucose 6 phosphate dehydrogenase protein (G6PD), GI: 238031000 was compared with AOL v16 and with NCBI Protein on 17^th^ May, 2016.

| **AOL full FASTA3** | Low scoring matches were identified with *E* scores ranging from 0.91 to 9.4 for 6 proteins that had identity matches of 24% to 47% over short alignments of 88 down to 30 AA. These proteins were not homologues and the identity matches appear random. |
| --- | --- |
| **AOL 80mer FASTA3** | The only alignment with >35% identity over 80 AA was to the putative allergen, *Blattella germanica*, German cockroach, Bla g 3 (hemocyanin) with 37% identity as the best aligned 80 AA segment. Bla g 3 only had 25% identity over full alignment with #5b protein with a highly insignificant *E* score of 33. |
| **AOL Exact 8 AA** | No exact 8 AA matches were identified to any protein in AOL v16. |
| **NCBI BLASTP**  **Allergen** | No significant identity match was identified in NCBI with keyword allergen. |
| **NCBI BLASTP**  **Toxin or Toxic** | Many significant (very small E score) alignments were identified (as low as 6e-93 for toxin, 6e-95 for toxic) to homologues from organisms noted for toxins or toxicity (e.g. *Bacillus sp*., *Enterococcus faecalis*, *Streptomyces sp*., *Clostridium sp*.). The alignments were approximately 35% for proteins identified with “toxin”, and 35% as the highest for those identified with “toxic” organisms (*Fictibacillus phosphorivorans*). The enzyme is common to all organisms. It is not a toxin and is not toxic to mammalian cells. |
| **NCBI BLASTP**  **No keyword** | Many high scoring identity matches were found with homologous proteins from other yeasts and molds. The full-length of #5b alignment to *Saccharomyces sp*. was with an E score of 0.0 and identity of 64% over 495 AA. |
| **Conclusion:** There does not seem to be a risk of allergy based on the lack of alignment to allergens beyond the low-level alignment to Bla g 3. Many homologues were identified to proteins from yeasts, including *Saccharomyces sp*. There are modest alignments to proteins that have not been demonstrated to be toxins, but are from organisms that are known to be toxic.  Therefore, risks from consumption of this protein appear to be minimal. | |

**Table S13 Bioinformatics Summary Results of Protein Band #5c.** The 525 AA hypothetical protein (PAS), GI: 238031215 was compared with AOL v16 and with NCBI Protein on 17^th^ May, 2016.

| **AOL full FASTA3** | Two very significant scoring alignments were identified, one with an *E* score of 5e-126 and 58% identity with Cla h 10 (*Davidiella sp.* aldehyde dehydrogenase) and one with 1e-124 *E* score and 58% identity to Alt a 10 (*Alternaria alternata* aldehyde dehydrogenase). Those proteins are minor hypothetically airway allergens identified only by low-level IgE binding in the studies used to identify them as putative allergens. The next highest alignments were to diverse species and of much greater *E* scores and lower identities. |
| --- | --- |
| **AOL 80mer FASTA3** | Three alignments were found with >35% identity. The highest scoring ones were to Cla h 10 and Alt a 10 with highest scoring 80mers of 72% identity. The third alignment was a storage mite, *Lepidoglyphus destructor* Lep d 13 with best identity of 35%, which only had 29.6% identity over full alignment with #5c protein with a highly insignificant *E* value of 96. |
| **AOL Exact 8 AA** | A number of exact 8 AA matches were identified to the Cla h 10 protein and Alt a 10 protein. |
| **NCBI BLASTP**  **Allergen** | There were a few significant alignments using NCBI with keyword allergen. The best scoring identities were 58-59% over full length to Cla h 10 and Alt a 10, two mold allergens. |
| **NCBI BLASTP**  **Toxin or Toxic** | Some significant (very small *E* score) alignments were identified (as low as 2e-171 for toxin, 2e-177 for toxic) to homologues from organisms noted for toxins or toxicity (e.g. *Bacillus sp*.). The alignments were approximately 53% for proteins identified with “toxin”, and “toxic” organisms. The enzyme is common to all organisms. It is not a toxin and is not toxic to mammalian cells. |
| **NCBI BLASTP**  **No keyword** | Many high scoring identity matches were found with homologous proteins from other yeasts and molds. The full-length PAS alignment to *Saccharomyces sp*. was with an *E* score of 0.0 and identity of 69% over 512 AA. |
| **Conclusion:** There does not seem to be a risk of allergy based on the lack of alignment to allergens. There are modest alignments to proteins that have not been demonstrated to be toxins, but are from organisms that are known to be toxic. Many homologues were identified to proteins from yeasts, including *Saccharomyces sp*. Therefore, risks from consumption of this protein appear to be minimal. | |

**Table S14 Bioinformatics Summary Results of Protein Band #6.** The 501 AA mitochondrial aldehyde dehydrogenase protein, GI: 238033249 was compared with AOL v16 and with NCBI Protein on 17^th^ May, 2016.

| **AOL full FASTA3** | Cla h 10 and Alt a 10 were the two matches with *E* scores of 2.4e-138 and 1.2e-131 and identities of 60% and 57%. They are minor hypothetically airway allergens identified only by low-level IgE binding in the studies used to identify them as putative allergens [5]. |
| --- | --- |
| **AOL 80mer FASTA3** | Cla h 10 and Alt a 10 were identified in alignments with best-scoring identities of 76% and 74%. |
| **AOL Exact 8 AA** | Cla h 10 and Alt a 10 had a large number of 8 AA matches as expected based on high identity matches. |
| **NCBI BLASTP**  **Allergen** | High scoring mitochondrial aldehyde dehydrogenase fungal proteins were identified in NCBI as the clear alignments using keyword allergen. *Cladosporium* (*Davidiella sp*.) and *Aureobasidium namibiae* were highest scoring with *E* scores of 0.0 and identities of 61% and 60%. These proteins are putative allergens, IgE binding, no biological activity (*e.g*. BHR, SPT). |
| **NCBI BLASTP**  **Toxin or Toxic** | Slightly lower scoring alignments (compared to the fungal mitochondrial aldehyde dehydrogenases) or 6e-170 and 4e-165 and identity scores of 53% and 51% respectively were found for toxin. Interestingly, a human mitochondrial aldehyde dehydrogenase was the highest scoring protein with “toxic” as a search term, having an *E* score of 6e-173 and 52% identity. The next scoring match was to aldehyde dehydrogenase of *Bacillus thuringiensis* were identified using toxin and toxic keywords. The matches were to homologous proteins from bacteria (e.g. *Bacillus thuringiensis*) that do not have mitochondria, but the function is still needed in these bacteria. There is no published evidence was found that any aldehyde dehydrogenase is a toxin. |
| **NCBI BLASTP**  **No keyword** | Many matches of high scoring identity with homologous proteins from other yeasts and molds were identified. The highest scoring alignment with a *Saccharomyces sp.* was with an *E* score of 0.0 and identity of 62% over 488 AA. |
| **Conclusion:** There is a very minor potential risk of allergy based on the identity alignments to two putative mold airway allergens. The enzyme is ubiquitous and there has not been proof that these proteins cause allergic disease, only that they can bind IgE from some airway sensitized individuals. There is a slightly better alignment to the homologous protein from *Saccharomyces sp*.  Therefore, risks from consumption of this protein appear to be minimal. | |

**Table S15 Bioinformatics Summary Results of Protein Band #7a.** The 341 AA Delta-aminolevulinate dehydratase protein, GI: 238033645 was compared with AOL v16 and with NCBI Protein on 17^th^ May, 2016.

| **AOL full FASTA3** | Very low-scoring alignments with *E* scores of 4 to 10 were identified to glucanase enzymes from diverse sources (e.g. rubber tree, grass pollen, cockroach and wheat). These alignments were not significant. |
| --- | --- |
| **AOL 80mer FASTA3** | No matches were identified with >35% identity in any 80 AA match with AOL v16. |
| **AOL Exact 8 AA** | No exact 8 AA matches were identified with AOL v16. |
| **NCBI BLASTP**  **Allergen** | Three short, very minor identity matches with protein segments from *Parasteatoda tepidariorum*, not a known allergen. |
| **NCBI BLASTP**  **Toxin or Toxic** | Modest scoring alignments were identified using toxin and toxic keywords. The matches of approximately 40% identity were to homologous proteins from bacteria. No published evidence was found that these proteins are toxins. |
| **NCBI BLASTP**  **No keyword** | Many matches of high scoring identity with homologous proteins from other yeasts and molds were identified. The highest scoring alignment with a *Saccharomyces sp*. was with an *E* score of 0.0 and identity of 76% over 340 AA. |
| **Conclusion:** It is unlikely that there is any risk of allergy for this protein. The same is true for toxicity. In addition, there are very good alignments to homologous proteins from many molds including *Saccharomyces sp*., with 76% identity over 340 AA. Therefore, risks from consumption of this protein appear to be minimal. | |

**Table S16 Bioinformatics Summary Results of Protein Band #7b.** The 350 AA mitochondrial alcohol dehydrogenase protein, GI: 238031179 was compared with AOL v16 and with NCBI Protein on 17^th^ May, 2016.

| **AOL full FASTA3** | One significant-scoring alignment was identified with an *E* score of 1.2e-108 and 76% identity over the full- length (350AA) to *Candida albicans* Can a 1.0101. This protein should be considered a putative allergen as one publication demonstrated IgE binding to the apparent band of a partially purified protein [9] and was also expressed as a recombinant protein with very light IgE binding (compared to the native) by a pool of 4 sera [10], which had not absolutely demonstrated that the large number of subjects bound IgE to this specific protein. There was no demonstration of biological activity. |
| --- | --- |
| **AOL 80mer FASTA3** | Only the single protein Can a 1.0101 identified by full-FASTA was identified with >35% identity in any 80 AA match with AOL v16. |
| **AOL Exact 8 AA** | Only Can a 1.0101 contained exact 8 AA matches compared to protein band #7b. |
| **NCBI BLASTP**  **Allergen** | Two high identity matches (*E* scores 0.0, identities of 80% and 76%) were found to a dehydrogenase of *Candida albicans* as a putative allergen. A third alignment was identified with an *E* score of 0.001 and identity of 28% over 120 AA to *Salmonella enterica* putative oxidoreductase. |
| **NCBI BLASTP**  **Toxin or Toxic** | Modest scoring alignments were identified using toxin and toxic keywords to alcohol dehydrogenase enzymes of bacteria that are associated with toxicity (*Corynebacterium ulcerans*, *Bacillus thuringiensis*, *B. cereus*, *Escherichia coli* and *Streptococcus sp*.). The *E* scores were small 1e-87, 2e-55 and percent identities 36% to 42%. However, no published evidence was found that these proteins are toxins. |
| **NCBI BLASTP**  **No keyword** | Many matches of high scoring identity with homologous proteins from other yeasts and molds were identified. The highest scoring alignment with a *Saccharomyces sp.* was with an *E* score of 0.0 and identity of 74% over 347 AA. |
| **Conclusion:** It is unlikely that there is any risk of allergy or toxicity for this protein. In addition, there are very good alignments to homologous proteins from many molds including *Saccharomyces sp.* Therefore, risks from consumption of this protein appear to be minimal. | |

**Table S17 Bioinformatics Summary Results of Protein Band #7c.** The 342 AA malate dehydrogenase, GI:238034064 protein was compared with AOL v16 and with NCBI Protein on 17^th^ May, 2016.

| **AOL full FASTA3** | *Malassezia furfur* Mala f 4.0101 was identified with an *E* score of 4.2e-54 and 51% identity over the full-length (341AA). Mala f 4 should be considered a putative allergen as the publication demonstrating IgE binding to a natural, semi-purified protein, but there was another isoform isolated and two other proteins from the partially purified Mala f 4 [11]. There was no demonstration of biological activity of this protein. |
| --- | --- |
| **AOL 80mer FASTA3** | Two proteins were identified with >35% identities. One was as expected, Mala f 4.0101, with a highest identity score of 70%. The second was to Pis s 2, the convicilin of *Pisum sativum* with 36.2% identity as the best match. However, Pis s 2 only had 31% identity with *E* score of 45 to protein #7c over full FASTA3. |
| **AOL Exact 8 AA** | Only Mala f 4.0101 contained exact 8 AA matches compared to protein band #7c with AOL v16. |
| **NCBI BLASTP**  **Allergen** | The only match by BLASTP was to Mala f 4.0101 with an *E* score of 6e-71 and an identity of 45%. |
| **NCBI BLASTP**  **Toxin or Toxic** | Significant scoring alignments were identified using toxin and toxic keywords to the malate dehydrogenase enzyme. The highest scoring hit was to a rat (*Rattus norvegicus*) with *E* score 2e-94 and 47% identity. Then a number of toxic organisms (*Vibrio cholera* and *E. coli*) with slightly lower identities and slightly higher *E* scores. |
| **NCBI BLASTP**  **No keyword** | Many matches of high scoring identity with homologous proteins from other yeasts and molds were identified. The highest scoring alignment with a *Saccharomyces sp.* was with an *E* score of 2e-115 and identity of 57% over 346 AA. |
| **Conclusion:** It is unlikely that there is any risk of allergy for this protein as only one putative dermal allergen had a significant match. The risk of toxicity is similarly low. In addition, there are very good alignments to homologous proteins from many molds including *Saccharomyces sp.* Therefore, risks from consumption of this protein appear to be minimal. | |

**Table S18 Bioinformatics Summary Results of Protein Band #7d.** The 328 AA putative protein, GI:238033788 was compared with AOL v16 and with NCBI Protein on 17^th^ May, 2016.

| **AOL full FASTA3** | One low-scoring alignment was identified with an *E* score of 0.23 and 24.6% identity over the full-length (328AA) to *Juniperus occidentalis* Jun o 4.0101. This alignment is very low scoring and unlikely to be relevant to risks. Besides, there was no demonstration of biological activity. |
| --- | --- |
| **AOL 80mer FASTA3** | No alignments with >35% identity over any 80 AA match were identified. |
| **AOL Exact 8 AA** | No exact 8 AA match was identified to protein band #7d. |
| **NCBI BLASTP**  **Allergen** | No significant match was identified using BLASTP and keyword Allergen. |
| **NCBI BLASTP**  **Toxin or Toxic** | Modest scoring alignments were identified using toxin and toxic keywords to the malate dehydrogenase enzyme. The highest scoring hit was to a bacterial protein (*Escherichia coli* aldehyde oxidase) with *E* score 2e- 39 and 32% identity. Then a number of toxic organisms (*E. coli*, *Microcystis aeruginosa*, *Raphidiopsis brookii*) with slightly lower identities and slightly higher *E* scores. |
| **NCBI BLASTP**  **No keyword** | Many matches of very high scoring identities with homologous proteins from other yeasts and molds were identified with identities from 42% to 86%. |
| **Conclusion:** It is unlikely that there is any risk of allergy for this protein. The risk of toxicity is similarly low. In addition, there are very good alignments to homologous proteins from many molds including *Saccharomyces sp.* Therefore, risks from consumption of this protein appear to be minimal. | |

**Table S19 Bioinformatics Summary Results of Protein Band #8.** The 248 AA Triose Phosphate isomerase protein, GI: 238032989 was compared with AOL v16 and with NCBI Protein on 17^th^ May, 2016.

| **AOL full FASTA3** | Four high-scoring alignments were identified with *E* scores smaller than 9.3e-51 and 50-53% identities over nearly the full length of four triosephasphate-isomerase proteins of diverse sources (wheat Tri a 31, two isoforms of Der f 25 from house dust mite, and shrimp Cra c 8). Yet triosephosphate isomerases are ubiquitous and there is no published evidence for such wide-spread cross-reactivity of this enzyme. Thus, these alignments are unlikely to represent a risk of possible cross-reactivity. |
| --- | --- |
| **AOL 80mer FASTA3** | The same four proteins were identified. The highest alignment was 62% identity. |
| **AOL Exact 8 AA** | A number of exact 8 AA matches were identified with the same four proteins. |
| **NCBI BLASTP**  **Allergen** | Similar identity matches (51-52% over full-lengths) were found by BLASTP to triosephosphate isomerase of house dust mites and scabies. However, there is no evidence these proteins are cross-reactive with the Pichia protein. |
| **NCBI BLASTP**  **Toxin or Toxic** | Moderately high scoring alignments were identified using toxin and toxic keywords. The matches with *E* scores of 2e-55 to 7e-65 and identities of 40% to 50% were found to homologous proteins from bacteria (e.g. *Escherichia coli*, *Bordetella sp*., *Clostridium sp.*). No published evidence was found that these proteins are toxins. |
| **NCBI BLASTP**  **No keyword** | Many matches of very high scoring identities with homologous proteins from other yeasts and molds were identified. The highest scoring alignment with a *Saccharomyces sp.* was with an *E* score of 1e-128 and identity of 71% over 248 AA. |
| **Conclusion:** It is unlikely that there is any risk of allergy for this protein. The same is true for toxicity. In addition, there are very good alignments to homologous proteins from many molds including *Saccharomyces sp.* Therefore, risks from consumption of this protein appear to be minimal. | |

**Table S20 Bioinformatics Summary Results of Protein Band #9a.** The 161 AA hypothetical protein, GI:328350030 (cyclophilin) was compared with AOL v16 and with NCBI Protein on 17^th^ May, 2016.

| **AOL full FASTA3** | Seven relatively high-scoring alignments were identified with *E* scores smaller than 1e-46 to cyclophilin proteins of different sources (Fungus Asp f 27, House dust mite Der f 29, Yeast Mala s 6, unassigned allergen from plant *Catharanthus*, Birch Bet v 7, unassignmed allergen from carrot *Daucus*, and Fungus Asp f 11. Yet cyclophilins are ubiquitous and while there is evidence of *in vitro* cross-reactivity, there is no published evidence for such wide-spread clinical cross-reactivity. Thus, these alignments are unlikely to represent a risk of possible clinical cross-reactivity. |
| --- | --- |
| **AOL 80mer FASTA3** | The same seven proteins were identified as having alignments with >35% identity. |
| **AOL Exact 8 AA** | All of the same seven proteins had 8 AA matches. |
| **NCBI BLASTP**  **Allergen** | A few cyclophilins were identified with high matches (71% identity) of diverse allergenic sources (e.g. mold, *Aspergillus fumigatus* and *Malazessia furfur*; mites, *Suidasia medanensis* and *Dermatophagoides farina*;liver fluke, *Clonorchis sinensis*. However, there is not clear published proof that these proteins are clinically cross- reactive from such diverse sources. |
| **NCBI BLASTP**  **Toxin or Toxic** | Modest scoring alignments were identified using toxin and toxic keywords. The matches with *E* scores of 3e- 32 or larger and identity scores of approximately 40% to 50% identities to homologous proteins from bacteria (e.g. *Enterococcus sp*., *Legionella sp*., *Corynebacterium sp*., also with some unusual species in terms of toxicity, such as *Danio rerio*). No published evidence was found that these proteins are toxins. |
| **NCBI BLASTP**  **No keyword** | Many matches of very high scoring identities with homologous proteins from other yeasts and molds were identified. The best alignment was from *Saccharomyces sp.* with *E* score of 9e-88 and identity of 74% over 162 AA. |
| **Conclusion:** Cyclophilins are unlikely to pose a risk of allergy, although there can be some shared IgE binding. Toxicity is unlikely, as well since there are very good alignments to homologous proteins from many molds including *Saccharomyces sp.* Therefore, risks from consumption of this protein appear to be minimal. | |

**Table S21 Bioinformatics Summary Results of Protein Band #9b.** The 154 AA cytosolic superoxide dismutase protein, GI: 238034030 was compared with AOL v16 and with NCBI Protein on 17^th^ May, 2016.

| **AOL full FASTA3** | Twenty three isoforms of olive pollen superoxide dismutase Ole e 5 were aligned with nearly identical scores relative to this *P. pastoris* protein #9b. Ole e 5 was first characterized by immunoblotting in 1998 by Boluda [12], and Butteroni demonstrated IgE binding using sera from a large number of olive pollen allergic subjects in 2005 [13], but till now this allergen has only been characterized by IgE tests without publication demonstrating biological activity [14]. |
| --- | --- |
| **AOL 80mer FASTA3** | The same 23 were identified as having alignments with >35%. The highest alignment was 87% and all had 80 identities greater than 78%. |
| **AOL Exact 8 AA** | All of the same 23 proteins had 8 AA matches. |
| **NCBI BLASTP**  **Allergen** | Multiple isoforms of olive pollen (Ole e 5) were identified with *E* scores ranging from 3e-55 to 9e-55 and approximately 57% identity over 152 AA. As noted above, there is evidence of IgE binding to the olive pollen using olive-pollen allergic subjects, but no demonstration of direct biological reactivity. |
| **NCBI BLASTP**  **Toxin or Toxic** | The matches were identified to homologous proteins from bacteria (e.g. *Clostridium sp*., *Corynebacterium sp*.), with high identities and significant *E* scores. However, the linkage appeared to be due the ability of the homologous proteins in a wide array of organisms to de-toxify free radicals. No published evidence was found that these proteins are toxins. |
| **NCBI BLASTP**  **No keyword** | High scoring identity matches with homologous proteins from other yeasts and molds were identified. The highest scoring alignment with a Saccharomyces sp. with 79% identity over 154 AA. |
| **Conclusion:** The protein #9b, superoxide dismutase of *Pichia pastoris* is unlikely to pose a risk of allergy. The same is true for toxicity. In addition, there are very good alignments to homologous proteins from many molds including *Saccharomyces sp*.  Therefore, risks from consumption of this protein appear to be minimal. | |

**Table S22 Bioinformatics Summary Results of Protein Band #10.** The 84 AA hypothetical protein (mitochondrial ATPase inhibitor) was compared with AOL v16 and with NCBI Protein on 17^th^ May, 2016.

| **AOL full FAST A3** | Relatively low-scoring alignments were identified with *E* scores between 0.1 and 10 and identities of 19-30% that were primarily identified to the muscle protein, tropomyosin. The alignments are clearly poor and do not represent conservation of homology. Thus, it is unlikely to represent a risk of cross-reactivity. |
| --- | --- |
| **AOL 80mer FASTA3** | No matches were identified with >35% identity in any 80 AA match. |
| **AOL Exact 8 AA** | No exact 8 AA matches were identified. |
| **NCBI BLASTP**  **Allergen** | No significant alignments were identified. |
| **NCBI BLASTP**  **Toxin or Toxic** | No significant alignments were identified. |
| **NCBI BLASTP**  **No keyword** | A few significant alignments were found for yeasts and molds. The highest scoring alignment with a  *Saccharomyces sp.* was with an *E* score of 3e-20 and identity of 62% over 64 AA. |
| **Conclusion:** The ATPase inhibitors do not appear to be allergens or toxins. The highest identity score to *Saccharomyces sp*. was with an *E* score of 3e-20 and 49% identity over 69 AA. Thus, it is very unlikely that this protein is an allergen or a toxin. The risks from consumption of this protein appear to be minimal. | |

**Figure S1 Coomassie Brilliant Blue Stained SDS-PAGE Gel Showing the Serial Dilution of *P. pastoris* Produced LegHb Starting from 200% to 2.5% of Total Protein.** Lane M, molecular weight marker; Lane 1, 200% of total protein (2.96 µg); Lane 2, 150% of total protein (2.21 µg); Lane 3, 100% of total protein (1.47 µg);

Lane 4, 80% of total protein (1.18 µg); Lane 5, 60% of total protein (0.88 µg); Lane 6, 40% of total protein

(0.59 µg); Lane 7, 20% of total protein (0.29 µg); Lane 8, 10% of total protein (0.15 µg); Lane 9, 5% of

total protein (0.075 µg); Lane 10-11, 2.5% of total protein (0.037 µg).


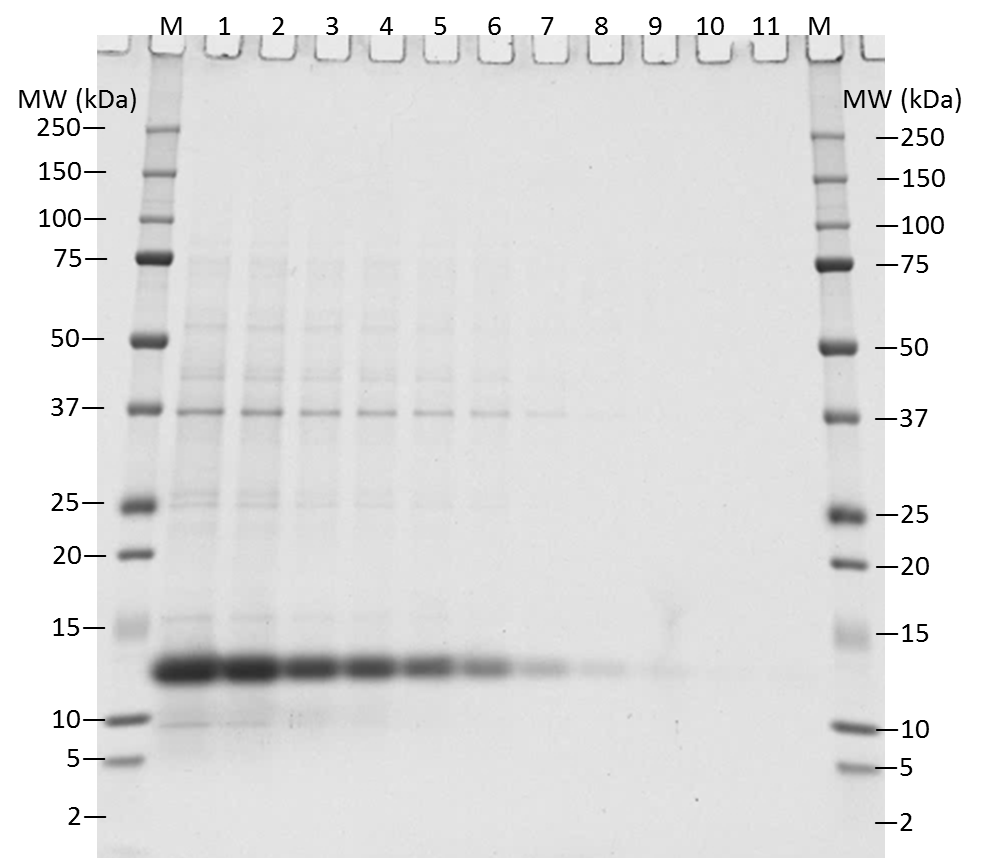


# Supporting references

[1] Banerjee, H. n., Verma, M., Search for a novel killer toxin in yeast *Pichia pastoris*. *Plasmid* 2000, *43*, 181-183.

[2] Chessa, R., Landolfo, S., Ciani, M., Budroni, M.*, et al.*, Biotechnological exploitation of Tetrapisispora phaffii killer toxin: heterologous production in *Komagataella phaffii* (*Pichia pastoris*). *Applied Microbiology and Biotechnology* 2017, *101*, 2931-2942.

[3] Comitini, F., Mannazzu, I., Ciani, M., Tetrapisispora phaffii killer toxin is a highly specific beta-glucanase that disrupts the integrity of the yeast cell wall. *Microbial cell factories* 2009, *8*, 55.

[4] Assarehzadegan, M. A., Sankian, M., Jabbari, F., Tehrani, M.*, et al.*, Identification of methionine synthase (Sal k 3), as a novel allergen of *Salsola kali* pollen. *Molecular Biology Reports* 2011, *38*, 65-73.

[5] Lauble, H., Kennedy, M., Emptage, M., Beinert, H., Stout, C., The reaction of fluorocitrate with aconitase and the crystal structure of the enzyme-inhibitor complex. *Proceedings of the National Academy of Sciences* 1996, *93*, 13699-13703.

[6] Chiu, L. L., Lee, K. L., Lin, Y. F., Chu, C. Y.*, et al.*, Secretome analysis of novel IgE‐binding proteins from *Penicillium citrinum*. *PROTEOMICS-Clinical Applications* 2008, *2*, 33-45.[7] Galli, B. D., Martin, J. G. P., da Silva, P. P. M., Porto, E., Spoto, M. H. F., Sensory quality of Camembert-type cheese: Relationship between starter cultures and ripening molds. *International Journal of Food Microbiology* 2016, *234*, 71-75.

[8] Kitamoto, K., Cell biology of the Koji mold *Aspergillus oryzae*. *Bioscience, Biotechnology, and Biochemistry* 2015, *79*, 863-869.

[9] Shen, H., Choo, K., Tang, R., Lee, C.*, et al.*, Allergenic components of *Candida albicans* identified by immunoblot analysis. *Clinical & Experimental Allergy* 1989, *19*, 191-196.

[10] Shen, H.-D., Choo, K.-B., Lee, H.-H., Hsieh, J. C.*, et al.*, The 40‐kilodalton allergen of *Candida albicans* is an alcohol dehydrogenase: molecular cloning and immunological analysis using monoclonal antibodies. *Clinical & Experimental Allergy* 1991, *21*, 675-681.

[11] Onishi, Y., Kuroda, M., Yasueda, H., Saito, A.*, et al.*, Two‐dimensional electrophoresis of *Malassezia* allergens for atopic dermatitis and isolation of Mal f 4 homologs with mitochondrial malate dehydrogenase. *European Journal of Biochemistry* 1999, *261*, 148-154.

[12] Boluda, L., Alonso, C., Fernández-Caldas, E., Purification, characterization, and partial sequencing of two new allergens of *Olea europaea*. *Journal of Allergy and Clinical Immunology* 1998, *101*, 210-216.

[13] Butteroni, C., Afferni, C., Barletta, B., Iacovacci, P.*, et al.*, Cloning and expression of the *Olea europaea* allergen Ole e 5, the pollen Cu/Zn superoxide dismutase. *International Archives of Allergy and Immunology* 2005, *137*, 9-17.

[14] Esteve, C., Montealegre, C., Marina, M., García, M., Analysis of olive allergens. *Talanta* 2012, *92*, 1-14.
